# Enhancing Mn‐Activated Mechanoluminescence via Pressure‐Regulated Local Structure in Centrosymmetric BaZnOS for Dynamic Response Applications

**DOI:** 10.1002/advs.202511805

**Published:** 2025-09-12

**Authors:** Hao Wang, Bohao Zhao, Tingting Zhao, Mei Li, Shang Peng, Xuqiang Liu, Yanlong Chen, Jiao An, Sheng Jiang, Yue‐chao Wang, Chuanlong Lin, Wenge Yang

**Affiliations:** ^1^ Center for High Pressure Science and Technology Advanced Research Beijing 100193 China; ^2^ National Key Laboratory of Computational Physics Institute of Applied Physics and Computational Mathematics Beijing 100094 China; ^3^ Shanghai Synchrotron Radiation Facility Shanghai Advanced Research Institute Chinese Academy of Sciences Shanghai 201204 China

**Keywords:** dynamic response, high pressure, mechanoluminescence, structure properties

## Abstract

Self‐recoverable mechanoluminescence (ML), typically observed in non‐centrosymmetric luminescent materials, has recently been reported in centrosymmetric systems such as Mn‐activated BaZnOS. However, the underlying mechanism and structure‐luminescence relationship remain unclear, hindering the development of high‐performance optoelectronics. Here, it is demonstrated that Mn^2+^‐activated BaZnOS exhibits strong, reproducible, and self‐recoverable piezoelectrically activated ML emission at the GPa level. Under compression at 0.6 GPa s^−1^, the ML intensity exhibits 10‐fold enhancement from ambient pressure to 1.5 GPa, but weakens above 1.5 GPa. Interestingly, it shows an oscillatory ML emission with time scale of 110 ms at rates of 2.3–2.7 GPa s^−1^. The ML behavior is distinct from photoluminescence (PL) with time scale of 0.3–1.5 ms, which shows slight attenuation at 0–4 GPa, exhibits threefold boost at 4–12 GPa, and then weakens above 12 GPa. The analyzed results show that the distinctive ML and PL behaviors stem from pressure‐regulated local structure, significantly affecting the local piezoelectricity and defect traps. Additionally, Mn^2+^‐activated BaZnOS exhibits diverse dynamic responses in both temporal and spatial dimensions for potential optoelectronic applications.

## Graphical abstract

The mechanoluminescence (ML) intensity and dynamic response of centrosymmetric BaZnOS: Mn^2+^ are boosted via pressure‐induced local distortion. In situ spectroscopy and calculations unveil the symmetry‐breaking mechanism, enabling high‐sensitivity ML materials for advanced force sensing.

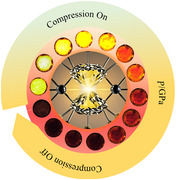

## 1 Introduction

Mechanoluminescence (ML), a light emission induced by mechanical stimuli, has garnered considerable attention due to its potential applications in structural diagnostics, artificial skins, bioimaging, and E‐signature systems.^[^ ^1^, ^2^, ^3^, ^4^, ^5^, ^6^, ^7^ ^]^ Among various types of ML, self‐recoverable ML stands out for its high sensitivity, reproducibility, and dynamic response to cyclic elastic deformation without damage.^[^ ^8^, ^9^, ^10^ ^]^ Unlike photoluminescence (PL), thermoluminescence (TL), and electroluminescence (EL), it does not require external voltage, UV light, or X‐ray irradiation, making it both environmentally friendly and highly versatile.^[^ ^11^, ^12^, ^13^ ^]^ The ML phenomenon has been observed in diverse materials including piezoelectrically‐activated ML in lanthanide/transition metal‐doped piezoelectrics (e.g., ZnS: Cu/Mn^2+^, CaZnOS: Mn^2+^/Ln^3+^),^[^ ^10^, ^14^, ^15^ ^]^ contact electrification‐induced ML in composite elastomers (e.g., Sr_3_Al_2_O_6_: Eu^3+^, Y_3_Al_5_O_12_: Ce^3+^),^[^ ^16^, ^17^, ^18^ ^]^ and ML in organic phosphors.^[^ ^19^, ^20^ ^]^ Despite significant progress in synthesizing multi‐color and multi‐mode ML materials^[^ ^1^ ^]^the impact of the lattice symmetry and local structure on ML properties remains poorly understood, limiting the development of high‐performance ML materials. Thus, elucidating the structural features that govern ML emission is crucial.

Lanthanide/transition metal‐doped piezoelectric materials with non‐centrosymmetric structures have been widely investigated for their exceptional luminescent properties.^[^ ^15^, ^21^, ^22^ ^]^ Unlike PL, whose mechanisms are well understood, the piezoelectrically activated ML involves a complex emission process. The widely accepted piezoelectric de‐trapping model suggests that the ML emission arises from the coupling effect of piezoelectric polarization, trapped carriers, band structures, and activators, all of which are influenced by the crystal structure of the host lattice and activator–host interactions.^[^ ^23^, ^24^ ^]^ Experimentally, it is commonly observed in the activator‐doped piezoelectric host materials with non‐centrosymmetric structure, e.g., lanthanide/transition metal‐doped ZnS, CaZnOS, and SrZnOS.^[^ ^15^, ^25^, ^26^, ^27^, ^28^ ^]^ The non‐centrosymmetric structures and activator‐host interactions significantly impact the piezoelectric polarization, trap distribution, and energy level of the activators,^[^ ^8^, ^29^ ^]^ determining self‐recoverable ML performance. This seemingly implies that self‐recoverable ML, which is piezoelectrically activated, can only occur in luminescent materials with non‐centrosymmetric structures. However, recent studies have reported self‐recoverable ML in centrosymmetric systems.^[^ ^16^, ^17^, ^30^ ^]^ For instance, BaZnOS has a centrosymmetric structure belonging to the *Cmcm* space group.^[^ ^27^ ^]^ It exhibits strong self‐recoverable ML behavior when doped with lanthanide/transition metal ions,^[^ ^15^, ^31^, ^32^ ^]^ which has been previously attributed to the local piezoelectricity due to the local asymmetry. Nevertheless, there is no experimental evidence to prove the local piezoelectricity and illustrate the relationship between the local structure and the ML property, essential for the exploration of high‐performance mechanoluminescent systems for optoelectronic applications within the centrosymmetric materials.

To date, the ML emission has been mainly studied at the MPa level using trial‐and‐error chemical methods such as altering the matrix, composites, categories, and concentration of the activators.^[^ ^21^, ^33^, ^34^ ^]^ Although these methods have successfully synthesized new ML materials, they offer limited insight into the structure‐ML relationship.^[^ ^35^, ^36^ ^]^ Pressure, as a powerful tool, can continuously tune the physical and chemical properties of materials by reducing atomic distances and enhancing host‐activator interactions,^[^ ^37^, ^38^, ^39^ ^]^ providing a deeper understanding of structure–property relationships. Recent studies have demonstrated that pressure can significantly alter the ML intensity and emission wavelength in non‐centrosymmetric materials such as Mn‐activated ZnS, SrZnOS, and SrZn_2_S_2_O, which exhibit rate‐dependent ML emission at the GPa level.^[^ ^40^, ^41^, ^42^ ^]^ However, it is still unclear how the structure affects the ML emission, and what kind of dynamic responses are applicable to potential optoelectronics in the pressure range from MPa to GPa. In this study, we investigate Mn‐activated ML in centrosymmetric BaZnOS at the GPa level and reveal pressure‐regulated ML behavior with rate‐dependent oscillatory emission characteristics. By a combination of various characterizations and the first‐principles calculations, we demonstrate that the evolution of the local structures significantly affects the ML property by modulating the polarization, defect traps, and band structure.

## 2 Results and Discussion

**Figure 1:**
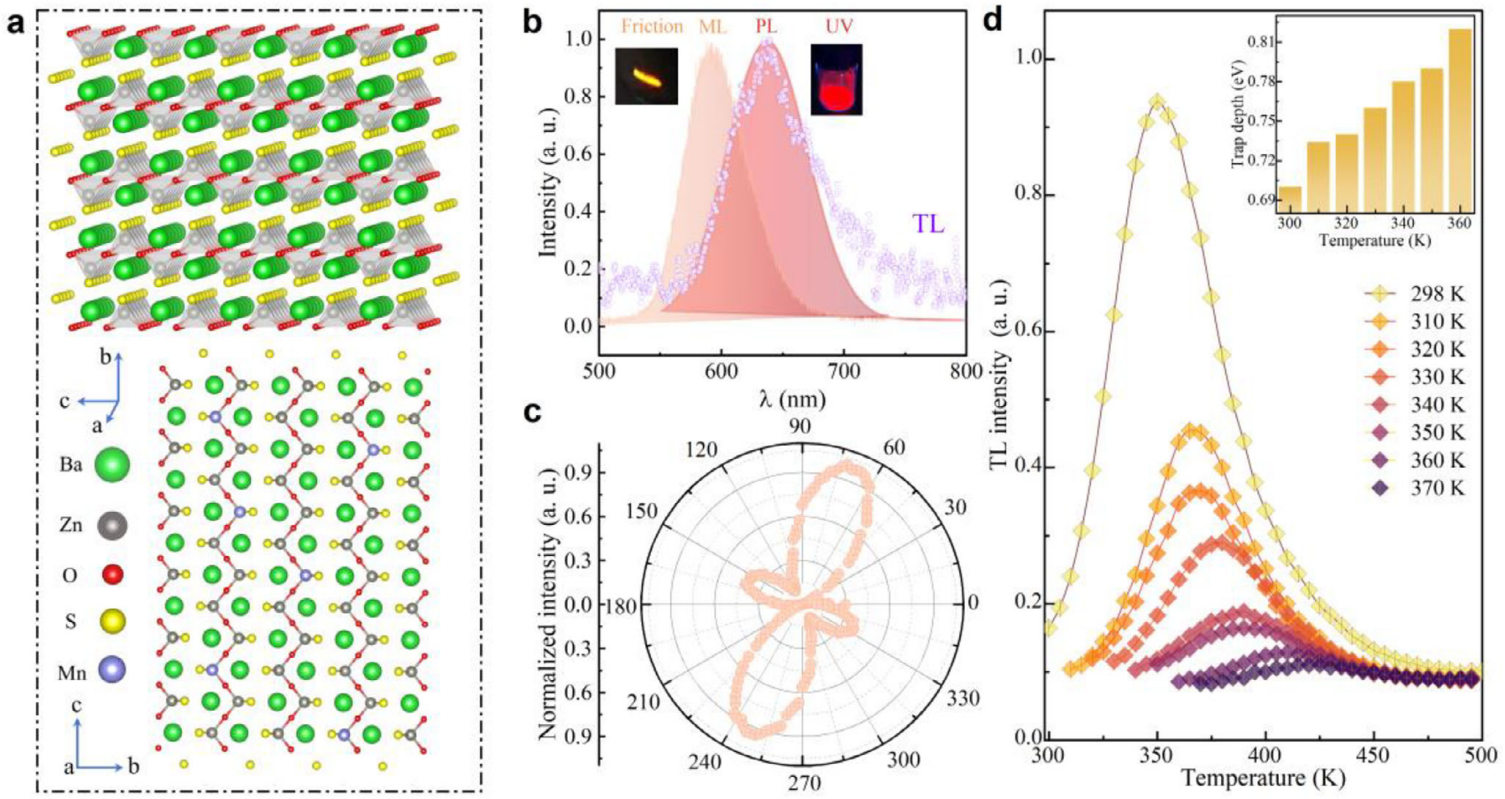
a) Schematic illustration of the crystal structure of BaZnOS. b) Comparison of the ML (orange), PL (red), and TL (blue cycle) spectra of BaZnOS: 0.01Mn^2+^. PL spectrum was excited by the laser of 405 nm. The inset shows PL and ML optical images induced by the UV and friction stimuli, respectively. The ML, PL, and TL spectra correspond to the electronic ^4^T_1_ to ^6^A_1_ transition of Mn^2+^. c) Polarized SHG signal of BaZnOS. d) Preheated temperature‐dependent TL property. The inset shows the trap depth.

2.1| Self‐Recoverable Mechanoluminescence Due to Locally Asymmetrical Structure in Centrosymmetric BaZnOS: Mn^2+^ BaZnOS crystallizes in a centrosymmetric orthorhombic structure (*Cmcm* space group, **Figure** **1**a).^[^ ^43^ ^]^ Each Zn^2+^ ion is tetrahedrally coordinated by two S^2−^ and two O^2−^ atoms. The ZnS_2_O_2_ tetrahedra share sulfur and oxygen vertices to form two sets of parallel S‐Zn‐…‐S‐Zn and O‐Zn‐…‐O‐Zn chains along the *a* and *c* axes, respectively, which interconnect to form puckered zinc oxysulfide layers separated by Ba^2+^ cations. The symmetric center is located at the oxygen site with the *C_2V_* point symmetry. On the other hand, the sulfur and oxygen atoms are arranged in opposite directions along the *b* axis, leading to the asymmetric tetrahedra. This local asymmetry may induce local polarization and nonlinear optical properties (e.g., piezoelectricity and second harmonic generation, SHG), typically associated with non‐centrosymmetric structures. In BaZnOS doped with luminescent activators, such piezoelectric polarization could further cause the de‐trapping and recombination of trapped carriers, leading to ML emission.

To investigate the influence of the local structure on the local polarization and luminescent properties, we synthesized pure and Mn^2+^‐doped BaZnOS powder samples by solid‐state reaction.^[^ ^32^, ^44^ ^]^ The structural characterizations of the as‐synthesized samples are shown in Figures S1 and S2 (Supporting Information). Under the excitation by the 405 nm laser and cyclic MPa‐level compression, Mn^2+^‐doped BaZnOS exhibits strong PL and reproducible ML, respectively, both visible to the naked eye (Figure 1b). Prior to ML measurements, samples were stored in darkness for several days without X‐ray or UV pre‐irradiation, confirming self‐recoverable ML emission. The ML peak at ≈590 nm shows a ≈45 nm blue shift compared to the PL peak at ≈635 nm, corresponding to the ^4^T_1_‐^6^A_1_ transition of Mn^2+^ (Figure S3, Supporting Information). The peak shift between ML and PL may be due to the effect of the locally asymmetric environment on the band of the Mn^2+^ ions,^[^ ^29^, ^45^ ^]^ as illustrated clearly below.

It is challenging to directly measure the local polarization in centrosymmetric structures and confirm the piezoelectric de‐trapping mechanism behind the self‐recoverable ML in BaZnOS: Mn^2+^. Here, we employed the SHG measurement to provide indirect evidence for the local polarization, as both SHG and piezoelectricity require structural asymmetry. SHG signals are detected in BaZnOS under ambient conditions across multiple orientations (Figure 1c, Figure S4, Supporting Information), suggesting the stress‐induced polarization due to the local asymmetry. In addition, we conducted the TL measurement (Figure 1d). The TL spectra is located at ≈636 nm (Figure 1b), and the peak of the TL curve shifts with increasing temperature. It shows that there are defect traps with the depth at 0.7‐0.82 eV for BaZnOS: Mn^2+^ and the TL intensity decreases at high temperatures. The combined SHG and TL results provide evidence for the existence of the local polarization and defect traps that are essential for the piezoelectricity and photoexcitation processes in the self‐recoverable ML.^[^ ^40^ ^]^

2.1

**Figure 2:**
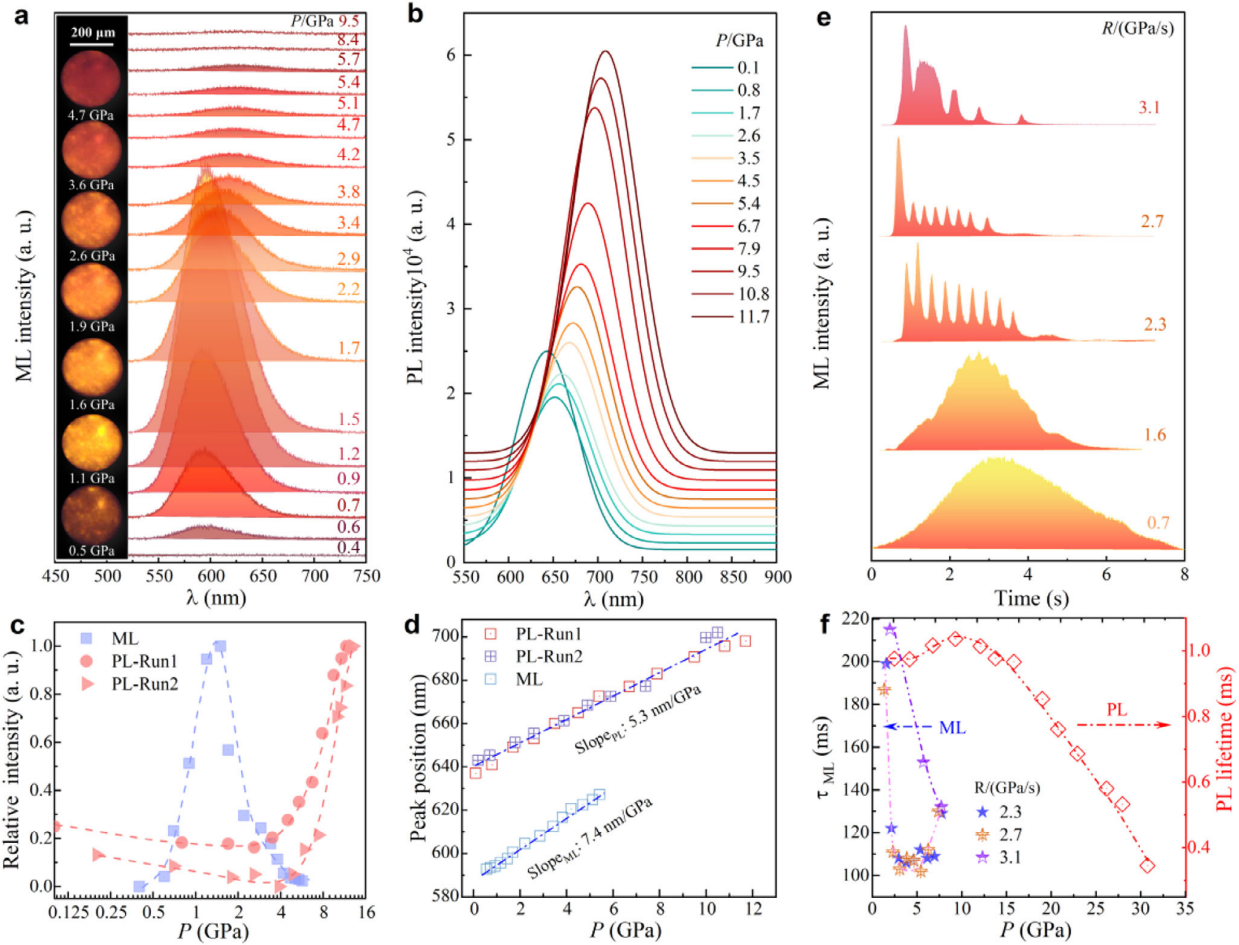
a) The ML spectra of BaZnOS: Mn^2+^ under compression at a rate of 0.3 GPa s^−1^ with microscopic optical images in inset. b) High‐pressure PL spectra of BaZnOS: Mn^2+^ excited by the laser of 405 nm. c) Emission intensity of BaZnOS: Mn^2+^ as a function of pressure. d) Pressure‐dependent peak position of PL and ML in BaZnOS: Mn^2+^ phosphor. e) Instantaneous ML intensity of BaZnOS: Mn^2+^ as a function of time under rapid compression at various compression rates. f) Comparison of PL decay time and ML emission characteristic time (*τ*_ML_). *τ*_ML_ is defined as the full width at half maximum of the oscillatory ML emission peaks.^[^ ^40^, ^41^ ^]^

2.2 | Pressure‐Regulated Mechanoluminescence with Temporal Characteristics under Rapid Compression We then study the ML emission of BaZnOS: Mn^2+^ at the GPa level. **Figure** **2**a shows the ML spectra under compression from ambient pressure to ≈10 GPa at a slow rate of 0.3 GPa s^−1^, compared to high‐pressure PL (Figure 2b and Figure S5, Supporting Information). As pressure increases, BaZnOS: Mn^2+^ emits the ML photon with the color varying from the orange to dark red (inset of Figure 2a). The wavelength shifts linearly from 592 nm at 0.6 GPa to ≈634 nm at 5.7 GPa with a slope of ≈7.4 nm per GPa (Figure 2d). The instantaneous emission intensity increases quickly from ambient pressure to ≈1.6 GPa with 10‐fold enhancement, and then drops slowly from ≈1.6 to 5.4 GPa after reaching a peak value. Above 6 GPa, the ML emission disappears. In contrast, the PL intensity decreases slightly under compression from 0.1 to ≈2.6 GPa, and then increases rapidly from 3.5 GPa to ≈12 GPa, ≈3 times higher than that at 0.1 GPa. This enhancement is also confirmed under the second pressure treatment (PL_run2 and Figure S6, Supporting Information). Under further compression above ≈12 GPa, the PL intensity becomes weaker and vanishes above ≈30.3 GPa. After pressure is released to 0.1 GPa, the PL intensity unexpectedly triples compared to its initial value (Figure S7, Supporting Information). Furthermore, the peak position has a large tunability with a red shift from 637 nm at 0.1 GPa to 785 nm at 30.3 GPa. At 0–12 GPa, it shifts linearly with the slope of ≈5.3 nm per GPa, smaller than that of ML. However, an anomalous shift occurs at 12–20 GPa (Figure S5, Supporting Information). The above results show that BaZnOS: Mn^2+^ exhibits distinctive ML and PL emission behaviors in terms of intensity and wavelength under high pressures.

Distinct from the PL process, the ML emission strongly depends on the compression rate and occurs under the mechanical stimulus on microsecond‐to‐second time scales. Therefore, it is necessary to investigate the ML characteristics under dynamic compression on different time scales. Figure 2e shows the instantaneous ML intensity as a function of time during the compression process at different rates, monitored by microsecond time‐resolved photomultiplier tube (PMT). Rate‐dependent distinct ML kinetics are revealed. Under slow compression at rates below 1.6 GPa s^−1^, broad emission peaks in the ML curve are observed. However, an oscillatory emission behavior with a series of sharp ML peaks appears at the critical rate of ≈2.3 GPa s^−1^, which is suppressed with the decrease in the intensity and the number of ML peaks above 3 GPa s^−1^. It has been confirmed by the time‐resolved fluorescence and ML imaging (Figure S8 and Movie S1, Supporting Information). The peak width and interval time between the adjacent ML peaks are ≈120 ms and ≈330 ms, respectively, two orders of magnitude longer than the lifetime of 0.3‐1.5 ms in the PL decay (Figure 2f and Figure S9, Supporting Information). Notably, the self‐recoverable ML emission likely involves intricate multi‐step processes and intermediate interactions. Thus, the characteristic timescale of piezoelectrically‐driven excitation and subsequent self‐recovery in dynamic response to mechanical stimuli should be different from the lifetime of PL that governs the exponential decay of PL intensity. The oscillatory ML behavior in BaZnOS: Mn^2+^ is similar to the cyclic photon emission reported previously in the piezoelectrically excited ML of Mn‐doped SrZnOS and SrZn_2_S_2_O, which is attributed to multi‐cyclic piezoelectricity and photoexcitation processes.^[^ ^40^, ^41^ ^]^ Therefore, we here attribute the oscillatory ML emission in BaZnOS: Mn^2+^ to the cyclic piezoelectric excitation process, as it follows the same ML mechanism, i.e., the piezoelectric de‐trapping model. The oscillatory ML behavior at the critical rate implies the intrinsic sub‐second temporal characteristics in the dynamic response to rapid compression.

The ML emission is related to the coupled effect of the piezoelectric polarization, defect traps, band structures, and activators. All these factors change as the crystal structure evolves with pressure. To understand the structure‐property relationship, in the following sections, we first investigate the evolution of the crystal structure under high pressures, as well as the changes in local polarization and defect traps. Combined with the first‐principle calculation, we then discuss how the pressure‐regulated crystal structure impacts the ML behavior.

**Figure 3:**
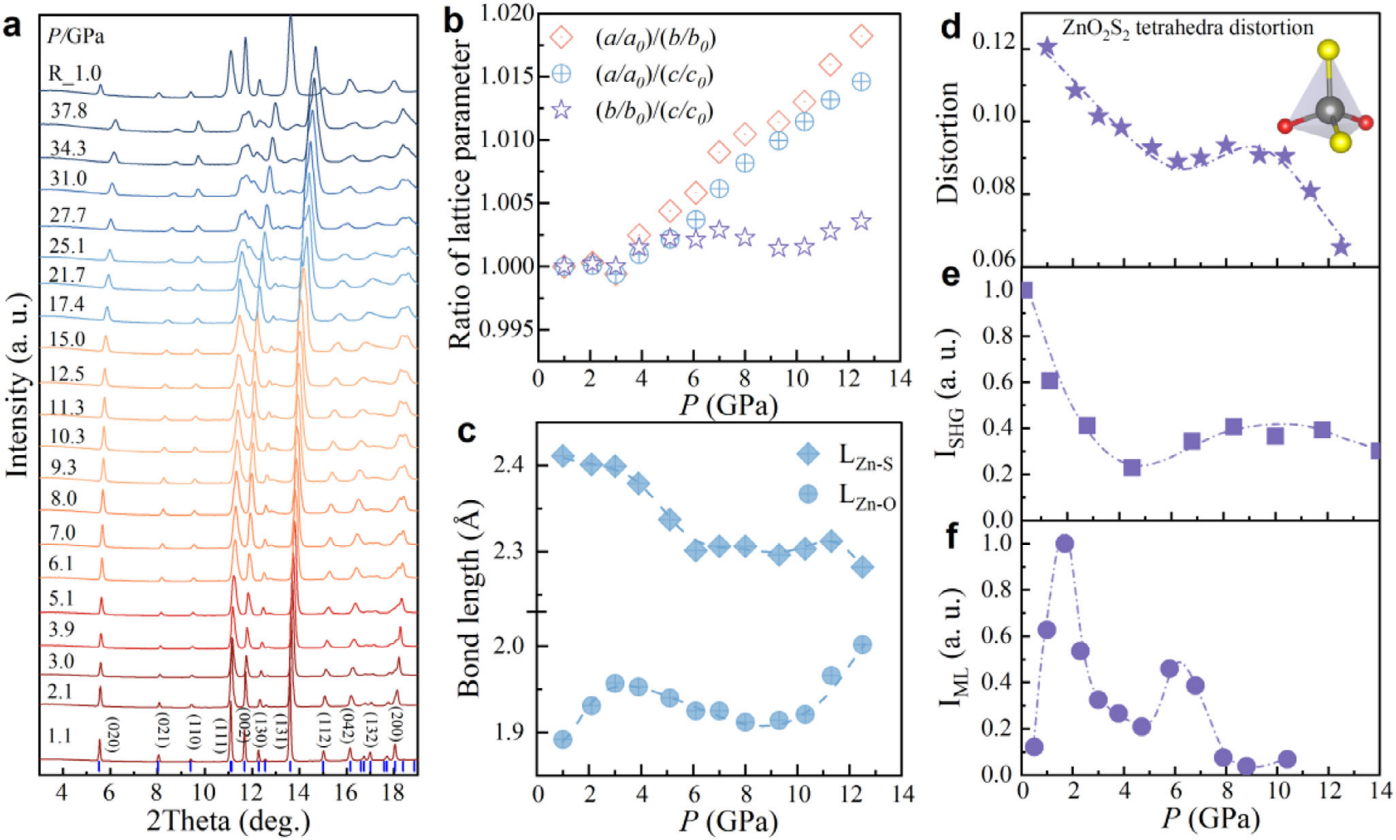
a) In situ high‐pressure synchrotron XRD patterns of Mn‐doped BaZnOS. b) Lattice parameter as a function of pressure. c) Bond length as a function of the pressure. d) Variation of the tetrahedral distortion with pressure. The inset graph is the structure of the ZnO_2_S_2_ tetrahedra. e) Pressure‐induced SHG signal response. f) Total ML intensity (*I*_onset_) at different onset pressures. The instantaneous ML intensities were collected by rapid compression of BaZnOS: Mn^2+^ from different onset pressures to 10 GPa at the rate of 15 GPa s^−1^. The compression rate is much higher than the critical rate, and therefore the self‐recoverable process has no time to occur.

2.3 | Pressure‐Regulated Crystal Structure with Alternation in Local Properties X‐ray diffraction (XRD) and Raman experiments were performed to investigate structural changes in BaZnOS under high pressure. Upon compression, BaZnOS maintains its centrosymmetric orthorhombic structure up to at least 15 GPa and displays a smooth reduction in lattice parameters (*a*, *b* and *c*) and unit cell volume (**Figure** **3**a,b and Figure S10, Supporting Information), indicating pressure‐induced contraction in the crystal lattice. High‐pressure Raman spectra further confirm the structure is stable at least up to 15 GPa (Figure S11, Supporting Information). The *a*, *b*, and *c* axes exhibit similar compressibility at ≈0–4 GPa. Above ≈4 GPa, *a* is less compressible than the *b* and *c* axes. Compared to the S─Zn─…─S─Zn chain along the *a*‐axis, it implies the O─Zn─…─O─Zn chain along the *c* direction has a similar compressibility at 0–4 GPa, but becomes more compressible above 4 GPa. The bond lengths were obtained by the refinement of the XRD patterns (Figure 3c, Figure S12 and Table S1, Supporting Information). The Zn─S bond length decreases at ≈0–6 GPa, and keeps a platform at ≈6–12 GPa. In contrast, the Zn─O bond increases slightly at 0–3 GPa, and then exhibits a slight decrease at ≈4–9 GPa, and finally increases above 9 GPa. To describe the distortion of the ZnS_2_O_2_ tetrahedra at high pressure, we define the distortion degree by using the formula:^[^ ^46^, ^47^, ^48^ ^]^

(1) $$D=\frac{1}{n}\;\sum_{i=1}^{n}\left(\left|L_{i}-L_{av}\right|/L_{av}\right)$$

where *D*, *n*, *L_i_*, and *L_av_* are distortion degree, number of bonds in the ZnS_2_O_2_ tetrahedra, the length of the Zn─S or Zn─O bonds, and the average bond length in the ZnS_2_O_2_ tetrahedra, respectively. Figure 3d shows that the distortion degree decreases rapidly at ≈0–6 GPa, then almost keeps flat at ≈6–9 GPa, and finally decreases rapidly above 9 GPa. Generally, this indicates that the ZnS_2_O_2_ tetrahedra becomes less distorted and asymmetric with increasing pressure. First‐principles calculations identify the Raman bands at 284 cm^−1^ and 300 cm^−1^ as Ag and B_1_g modes, respectively (Figure S13 and Table S2, Supporting Information).^[^ ^49^ ^]^ They mainly correspond to the bending and strengthening vibrations of the Zn─S bond along the *b* and *a*‐axes, respectively. The band at 300 cm^−1^ shifts to a higher value with a larger slope than the band at 284 cm^−1^ (Figure S11, Supporting Information). It means the evolution of the distorted tetrahedra at high pressures is due to the enhanced interaction between the puckered zinc oxysulfide layers.

The ML emission involves the piezoelectricity and photoexcitation processes. To understand the effect of the structural modification, the SHG measurement of BaZnOS was conducted to investigate the change of the local polarization at high pressures. Generally, the SHG intensity drops rapidly by 80% at 0.1–4 GPa, increases slightly at ≈4–8 GPa, and decreases again with pressure until it almost disappears at ≈15 GPa (Figure 3e). BaZnOS exhibits weak polarization above 4 GPa. The trend of the SHG at high pressures is consistent with the change of the tetrahedral distortion. This implies that the capability of the piezoelectrical polarization becomes weak quickly at 0–4 GPa due to the pressure‐regulated local structure, unfavorable for the piezoelectricity process of the ML emission.

Meanwhile, the ML intensities (*I*_onset_) at different onset pressures (*P*_o_) were measured to estimate pressure‐dependent effective trap density involving the ML kinetic process. BaZnOS: Mn^2+^ was compressed rapidly from different onset pressures to ≈10 GPa. The compression rate is fast enough that the trapped carriers undergo only one cycle of piezoelectrically‐induced de‐trapping process (Figure 3f and Figure S14, Supporting Information). *I*_onset_ should be proportional to the effective trap number at the onset pressure, as self‐recoverable process did not occur during the rapid compression process. It is found that *I*_onset_ increases by 10 times when *P*_o_ changes from 0.2 GPa to ≈1.7 GPa and then decreases quickly after reaching the peak value. It corresponds to the pressure‐induced enhancement of the ML emission (Figure 2a). At 5–8 GPa, *I*_onset_ presents a weak peak again, indicating a slight increase of the effective trap density. It corresponds to the anomalous change of the tetrahedral distortion (Figure 3d). Therefore, the pressure‐dependent ML emission is attributed to the influence of the pressure‐regulated local structure on the effective traps that involving the ML emission.

**Figure 4:**
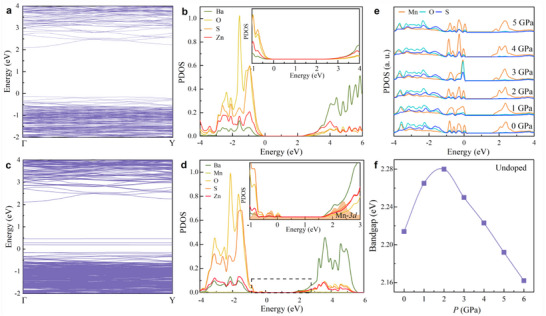
a) The band structure and b) PDOS of BaZnOS at ambient pressure. c) The band structure and d) PDOS of Mn‐doped BaZnOS at ambient pressure. e) The local PDOS of Mn at different pressures. f) The bandgap values of the pure BaZnOS as a function of pressure.

2.4 | Band Structures of Mn‐doped BaZnOS and Discussion on Structure‐Mechanoluminescence Relationship The density functional theory (DFT) method was employed to examine how structural modifications influence the band structure and electronic levels of Mn dopants in BaZnOS. The results show that Mn ions are successfully doped into centrosymmetric BaZnOS without significantly altering the host lattice and band structure (**Figure** **4**a–d). At ambient pressure, pure BaZnOS exhibits a direct bandgap of 2.21 eV, lower than the experimental value, as usually underestimated by the DFT calculations (Figure 4a).^[^ ^50^ ^]^ The projected density of states (PDOS) reveals that the valence band (VB) is dominated by the O‐2p and S‐3p states, while the conduction band (CB) primarily consists of Ba‐6s/5p states and Zn‐3d states (Figure 4b).^[^ ^43^, ^51^ ^]^ Specifically, the S and O states contribute significantly to the valence band below the Fermi level (Figure 4b). In Mn‐doped BaZnOS, the introduction of Mn dopants did not significantly affect the lattice structure and band structure of the matrix (Figure 4c,d), which is consistent with the experimental characterizations. However, the comparison of the local PDOS near Zn and Mn shows that the introduction of Mn leads to the existence of certain localized states near the Fermi levels and empty states of 3d‐orbitals of Mn^2+^ near the CB of Mn‐doped BaZnOS at ambient pressure, between which photon emission can take place.^[^ ^25^ ^]^ Moreover, the interelectronic levels are formed by the Mn dopants, which may facilitate the electron transfer to enhance the ML performance.

At high pressures, the theoretical calculations confirm that the doped and undoped BaZnOS exhibit stable *Cmcm* structure in the experimental pressure range. The lattice structure exhibits a continuous reduction without the phase transition with increasing pressures, consistent with the XRD measurement. The overall band structures for Mn‐doped BaZnOS did not change significantly at high pressures (Figure S15, Supporting Information). However, the bandgap of the matrix presents a maximum value at ≈1–2 GPa, namely, it increases first and then decreases above 2 GPa (Figure 4f and Figure S16, Supporting Information). It corresponds to the trend of the pressure‐dependent ML intensity, indicating that the change of the band structure affects the ML. On the other hand, the local PDOSs of Mn^2+^ reveal slight changes in the bands of Mn^2+^ near the CB and VB at 0–5 GPa (Figure 4e). We note that the unoccupied states of Mn^2+^ are split due to the locally asymmetric environment, consisting of at least two main energy levels. We speculate that the PL peak corresponds to the emission transition from the low energy levels of Mn^2+^ to the ground state, which can be directly excited by the laser, while the ML peak is from the high excited energy level of Mn^2+^, as the de‐trapped electrons in the conduction bands can readily transfer to the high energy levels of Mn^2+^.

Combined the experimental and calculated results, we then discuss how the pressure‐regulated structure affects the ML process. In Mn‐activated BaZnOS, the piezoelectrically‐activated ML process can be summarized as follows:^[^ ^37^, ^40^, ^41^ ^]^ under deviatoric stress, deformation‐induced polarization generates a local piezoelectric field, which releases trapped electrons and holes near Mn^2+^ activators. The escaped de‐trapped electrons are thermally activated into the CB. Under piezoelectrical perturbation, the CB is tilted due to the piezoelectric effect, promoting the recombination of the de‐trapped electrons and holes with the release of radiative energy. Then the Mn^2+^ ions are excited from the ground state (^6^A_1_) to the excited state (^4^T_1_), resulting in photon emission. Within the piezoelectrical de‐trapping model, the piezoelectrical field, effective traps, bandgap of the host lattice, energy levels of activators are crucial for the ML process. The effective traps and calculated bandgaps exhibit similar pressure dependence at 0–4 GPa, suggesting that the structural effect on traps may be mediated through bandgap changes. Specifically, the bandgap changes at 1.6 GPa promote carrier escape from traps. Above 1.6 GPa, these changes hinder the escape of carriers from the defect traps. In addition, the local polarization is reduced rapidly by 80% at 0.1–4 GPa, unfavorable for the piezoelectrically‐activated process. These results imply that the pressure‐regulated structure mainly affects the ML emission through the local polarization and effective traps, leading to a coupled effect on the piezoelectricity process. In contrast, the PL process arises from conventional photon excitation, where photon absorption promotes Mn^2+^‐3d electrons to excited states that subsequently decay radiatively. Under mechanical compression, both ML and PL display non‐monotonic intensity variations originating from pressure‐modulated distortions of the [Zn/MnS_2_O_2_] tetrahedral units. Remarkably, their emission spectra maintain identical characteristics, as both processes involve identical 3d–3d transitions of Mn^2+^ ions. The spectral features are exquisitely sensitive to the local crystal field environment, namely, increasing pressure strengthens the crystal field at [Zn/MnS_2_O_2_] tetrahedra. This reduces the ^4^T_1_→^6^A_1_ energy splitting and produces a systematic red‐shift. Moreover, it modifies the emission band shape through changes in electron–phonon coupling.^[^ ^52^, ^53^ ^]^ Hence, these results show that the pressure‐induced ML and photon‐excited PL are mainly affected by pressure‐enhanced interaction between the lattice and dopant and change of tetrahedral distortion.

**Figure 5:**
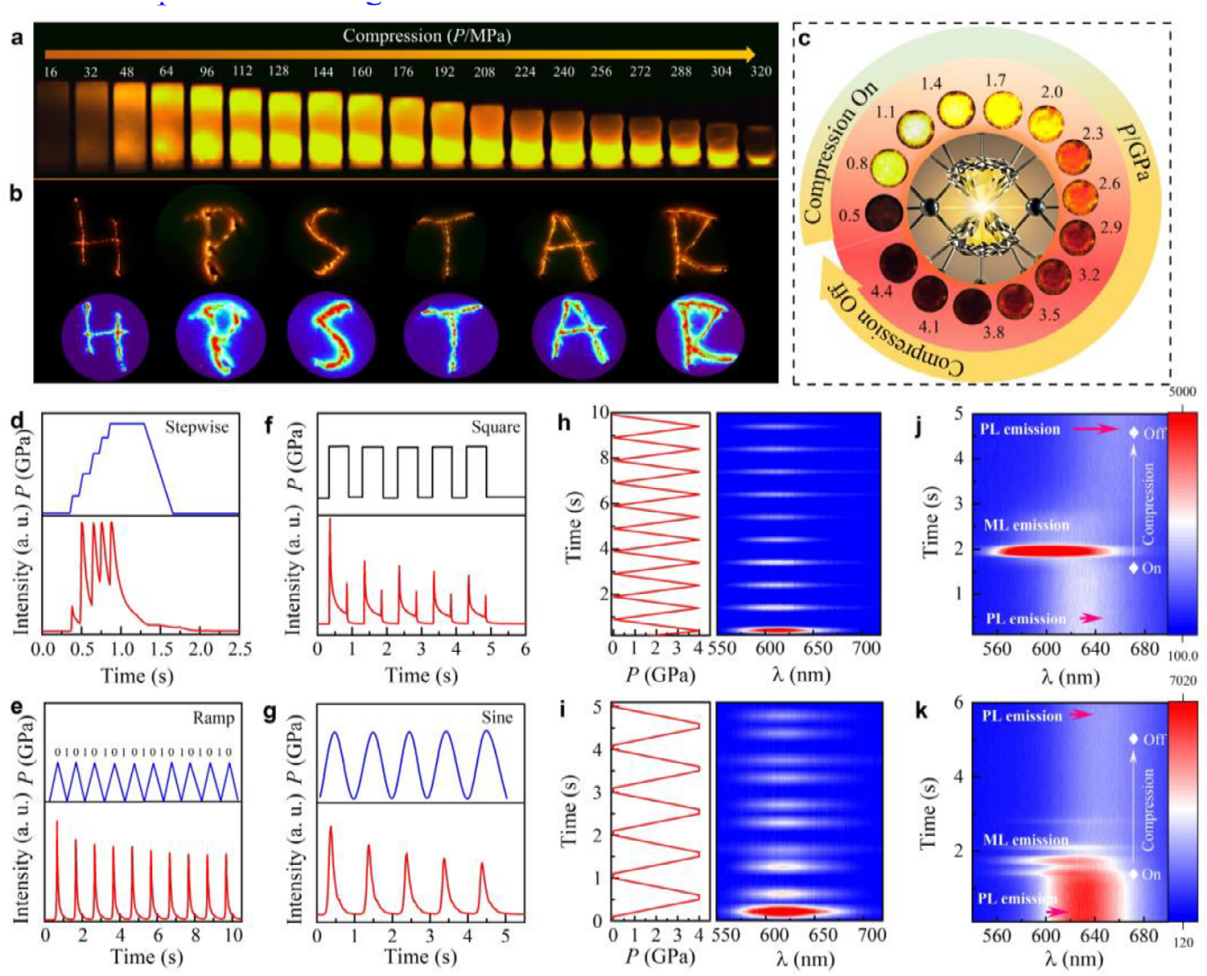
a) Dynamic stress visualization of BaZnOS: Mn^2+^ phosphor composite cylinder under MPa pressure. b) ML trajectory and stress distribution of the composite film when writing “HPSTAR”. c) Dynamic microscopic ML imaging at the GPa level. Dynamic ML response under d) stepwise, e) multi‐cyclic square, f) ramp and g) sine compression‐decompression profiles. h,i) Dynamic ML spectra for pressure sensor application. PL and ML responses under 405 nm laser irradiation and dynamic compression. j) Coexistence of the ML and PL emission under compression from ambient pressure to 5 GPa at a rate of 5.5 GPa s^−1^ and k) 2.3 GPa s^−1^. Here, the relatively low power of the 405 nm laser results in weak PL emission intensity, consequently enabling enhanced observation of the ML emission.

2.5 | Dynamic ML Response for Application Inspired by the multiple luminescence responses from MPa to GPa levels, we designed applications for micron‐scale dynamic ML, including microscopic imaging, multi‐mode stimulus‐response, and binary information storage. In **Figure** **5**a, the ML intensity distribution of an epoxy resin‐phosphor composite cylinder under various stresses demonstrates that stress magnitude and distribution can be visualized through ML intensities (Movie S2, Supporting Information). The film's ability to visualize dynamic stress was further tested by writing on it with a pen at room temperature (Figure 5b). The ML profile clearly traced the pen tip's trajectory, revealing handwriting patterns like “HPSTAR.” The quantitative relationship between ML intensity and stress makes relative ML brightness a key indicator of spatial stress information, enhancing security by incorporating stress‐related spatial features. In the GPa range, dynamic microscopic imaging shows that ML emission intensity changes from dark to bright and back to dark with increasing pressure, accompanied by a color shift from yellow to orange to red (Figure 5c). This behavior provides a significant reference for extreme‐condition pressure sensing. To investigate dynamic ML at GPa levels, four waveforms (stepwise, square, ramp, and sine) were used to drive the dDAC for ML signal collection (Figure 5d–g). The results show that instantaneous compressive pressure triggers electron de‐trapping and ML emission. The ML intensity increases with rising pressure and decreases when pressure stabilizes. Here, compression is denoted as “0” and decompression as “1” indicating high‐pressure sensitivity. During cyclic compression‐decompression, BaZnOS: Mn^2+^ phosphors emit periodic ML signals, analogous to binary codes (e.g., “01010101” for the letter “U”). The 2D ML spectral projection shows stable and reproducible emission spectra during these cycles (Figure 5h,i). Based on the distinct PL and ML characteristics and high stability of BaZnOS: Mn^2+^, we combined the dDAC with a 405 nm laser to design an encryption label (Figure 5j,k). Under 405 nm laser irradiation, the Mn^2+^ emission peak is at ≈630 nm. Upon dynamic pressure application, ML emission appears, and oscillatory ML can be achieved at the critical compression rate.

## 3 Conclusion

In summary, we systematically investigated the luminescence characteristics of centrosymmetric BaZnOS: Mn^2+^ via pressure‐regulated local structural. Continuous compression from ambient to 10 GPa demonstrated pressure‐dependent evolution of ML properties, showing remarkable emission enhancement in the mild pressure regime (1–2 GPa) accompanied by tunable emission color. In contrast, the PL intensity exhibited non‐monotonic pressure dependence, which shows slight attenuation at 0–4 GPa, exhibits three fold boost at 4–12 GPa, and then weakens above 12 GPa. Additionally, rate‐dependent oscillatory ML was observed within a critical rate range, attributed to multi‐cycle piezoelectric‐induced excitation and self‐recovery processes. Comprehensive analysis, including structural evolution, SHG response, trap distribution, and theoretical calculations, demonstrated that the distinct behaviors of ML and PL stem from pressure‐induced tetrahedral distortion, which affects local piezoelectric fields, defect trap states, and activator energy levels. Our findings highlight the material's potential for diverse applications in extreme environments (MPa‐GPa pressure range) and provide a novel approach to studying ML responses in centrosymmetric materials, elucidating the interplay between local structure, piezoelectric effects, and defect traps.

## 4 Experimental Section

Detailed experimental procedures are reported in Supporting Information.

## Conflict of Interest

The authors declare no conflict of interest.

## Supporting information



Supporting Information

Supplemental Data

## Data Availability

The data that support the findings of this study are available in the Supporting Information of this article.
