# Supplementary material for: Enhancing Mn‐Activated Mechanoluminescence via Pressure‐Regulated Local Structure in Centrosymmetric BaZnOS for Dynamic Response Applications
Source: Adv Sci (Weinh). 2025 Sep 12;12(45):e11805. doi: 10.1002/advs.202511805 (PMC12677602; doi:10.1002/advs.202511805)
Supplement: Supplementary file 1 — Supporting Information [file ADVS-12-e11805-s002.docx]

Supporting Information

**Enhancing Mn-activated mechanoluminescence via pressure-regulated local structure in centrosymmetric BaZnOS for dynamic response applications**

Hao Wang^1#^, Bohao Zhao^1,3#^, Tingting Zhao^1^, Mei Li^1^, Shang Peng^1^, Xuqiang Liu^1^, Yanlong Chen^1^, Jiao An^1*^, Sheng Jiang^2^, Yuechao Wang^3^, Chuanlong Lin^1*^ and Wenge Yang^1^

^1^Center for High Pressure Science and Technology Advanced Research, Beijing 100093, China

^2^Shanghai Synchrotron Radiation Facility, Shanghai Advanced Research Institute, Chinese Academy of Sciences, Shanghai 201204, China

^3^Laboratory of Computational Physics, Institute of Applied Physics and Computational Mathematics, Beijing, 100088, China.

*Corresponding author: [chuanlong.lin@hpstar.ac.cn](mailto:chuanlong.lin@hpstar.ac.cn), [jiao.an@hpstar.ac.cn](mailto:jiao.an@hpstar.ac.cn)

**Experimental section**

**Materials and Synthesis**: Mn-doped and undoped BaZnOS phosphors were prepared by a solid-state reaction method.^[1]^ The raw materials include BaCO_3_ (99.99%, Aladdin), ZnS (99.99%, Aladdin), and MnCO_3_ (99.99%, Aladdin). The reagents including the main raw materials of BaCO_3_ and ZnS with regulating agents of MnCO_3_ were all in analytical pure and directly used without any further purification.^[2]^ Typically, the reagents were firstly weighed stoichiometrically and thoroughly mixed/pulverized in an agate mortar with an appropriate amount of ethanol. Finally, the samples were synthesized at 1000 ◦C for ~3 h under N_2_ atmosphere (purity: 99.99%). Then, the sintered samples were naturally cooled down to room temperature and subsequently ground into powders for the following characterizations. In order to explore the ML performance under mechanical stimulus in the MPa range, two composites were prepared. The composite cylinder (20 mm in length and 6 mm in diameter) and composite films (≈5 mm in thickness) were prepared by mixing the phosphor powders in an optical epoxy resin (SpeciFix, Struers GmbH) at a particle-to-resin weight ratio of 1:5 and 1:1, respectively.

**Preparation of High-Pressure Samples**: The operational principle of the diamond-anvil cell (DAC) is elegantly straightforward: two single-crystal diamond anvils are aligned face-to-face so that their micro-scale culets oppose one another. Because pressure (P) is simply force divided by contact area, the exceptional hardness of diamond permits the application of large forces over the minute culet area—typically tens to hundreds of micrometres in diameter—thereby concentrating stress and generating static pressures that routinely exceed one million atmospheres on the specimen confined between the opposing culets. Symmetric DAC with a pair of 400 μm diameter culet-sized diamond anvils were used for all *in-situ* measurements under high pressure.^[3,4]^ A steel gasket (T301) was pre-indented to 30-40 μm in thickness and a 200 μm diameter hole was laser-drilled to serve as the sample chamber. The BaZnOS: Mn^2+^ and undoped BaZnOS powder samples were loaded in the chamber together with a small ruby ball for pressure determination by using the ruby fluorescence method.^[5]^ Silicone oil was used as a pressure-transmitting medium for high-pressure X-ray diffraction (XRD), Raman, and photoluminescence (PL) measurements. Mechanoluminescence (ML) experiments were conducted without any pressure medium.

***In situ* High-Pressure X-ray Diffractions (XRD)**. The *in-situ* high-pressure XRD experiments were conducted at the beamline BL15U1 of Shanghai Synchrotron Radiation Facility (SSRF) with the wavelength of 0.6199 Å and beam spot size of 5×9 *µ*m^2^. Two-dimensional diffraction images were collected continuously during the compression process with a PILATUS 2M detector, and CeO_2_ was used for standard calibration. The diffraction patterns were integrated into a one-dimensional profile by using the Dioptas software.^[6]^ Structure refinements were carried out using the Rietveld method to obtain the lattice parameters using General Structure Analysis software (GSAS).^[7]^

**High-Pressure Raman**: Raman spectra were recorded on a Renishaw Raman microscope using a 532 nm laser. The system was calibrated by the Raman signal of Si, and spectra were collected in the range of 100-1000 cm^-1^.

**Second harmonic generation (SHG) measurement**: SHG experiment was measured in a home-designed optical system (Ideaoptics, China). The high-pressure SHG measurement is based on the ambient powder SHG measurement extended by Kurtz and Perry.^[8]^

**Photoluminescence, Lifetime and Thermoluminescence Characterizations**. In situ high-pressure photoluminescent spectra were recorded using a home-designed spectroscopy system equipped with NOVA2S highly sensitive spectrometer in the 360–930 nm (Ideaoptics, China) band with 375 nm and 405 nm lasers as the excitation source. Thermoluminescence experiments were performed using a TL spectrometer (LTTL-3DS, RongFan).

**Microsecond Time-Resolved Mechanoluminescence Measurement.** To investigate pressure-induced mechanoluminescence (ML) behavior at the GPa level under varying ramp rates, an experimental setup capable of combining rapid compression device with in situ ML detection on short timescales is essential. In this work, we developed a time-resolved fluorescence system integrated with dynamic diamond anvil cells (dDACs) to capture ML signals across different timescales. The dDAC, driven by a piezoelectric actuator, enables rapid compression modulation with ramp rates spanning from 0.001 to ~600 GPa/s. The fluorescence detection system comprises a dispersive grating and a high-speed camera (Zyla 4.2 Plus, Andor) with a maximum frame rate of 27,000 fps. During rapid compression experiments, the pressure-induced ML signals were continuously monitored at predefined ramp rates. The optical photographs of ML and ML intensity were captured by a high-speed camera (G516-C, Revealer) and a photomultiplier tube (C8855, Hamamatsu Photonics) on microsecond time scale.

**Calculation Setup:** First-principles calculations were performed by the Vienna Ab initio Simulation Package (VASP) based on density functional theory (DFT). The projector augmented wave (PAW) method is implemented to describe the electronic structures.^[9-11]^ The generalized gradient approximation (GGA) of Perdew-Burke-Ernzerhof functionals were adopted to describe the exchange-correlation functional.^[12]^ The energy cut off for the plane wave basis expansion was set to 550 eV, and the force on each atom less than 1×10^-3^ eV/Å was set for convergence criterion of geometry relaxation. The self-consistent calculations applied a convergence energy threshold of 1×10^-8^ eV. Phonopy was employed to calculate the phonon properties of the system.^[13,14]^ The valence states of the elements were set as (5*s*, 5*p*, 6*s*) for Ba, (3*d*, 4*s*) for Zn, (2*s*, 2*p*) for O, and (3*s*, 3*p*) for S. In the Mn-doped system, the valence state of Mn was (3*d*, 4*s*). A k-point grid of 8×3×5 was adopted for the BaZnSO unit cell to effectively sample the Brillouin zone. When calculating the electronic structure of the doped supercell, a supercell model of 3×2×3 was performed. Subsequently, the k-point sampling grid for the supercell was set to 4×2×3.

**Table S1. Structural parameters obtained from the refinement for BaZnOS.**

| *P*(GPa) | 1.0 | 2.1 | 3.0 | 3.9 | 5.1 | 6.1 | 7.0 | 8.0 | 9.3 | 10.3 | 11.3 | 12.5 | 15.0 |
| --- | --- | --- | --- | --- | --- | --- | --- | --- | --- | --- | --- | --- | --- |
| *T* | 298 K | | | | | | | | | | | | |
| Space group | *Cmcm* | | | | | | | | | | | | |
| *a* (Å) | 3.958(5) | 3.929(5) | 3.915(8) | 3.903(4) | 3.891(5) | 3.882(9) | 3.875(6) | 3.870(1) | 3.860(3) | 3.852(7) | 3.848  (5) | 3.846  (6) | 3.832  (1) |
| *b* (Å) | 12.819(1) | 12.724(2) | 12.662(2) | 12.613(1) | 12.565(2) | 12.528(4) | 12.489(2) | 12.439(4) | 12.413(1) | 12.371(2) | 12.282(2) | 12.263(2) | 12.111(6) |
| *c* (Å) | 6.103(1) | 6.059(7) | 6.035(1) | 6.019(9) | 5.993(8) | 5.971(1) | 5.952(6) | 5.929(1) | 5.907(4) | 5.886(7) | 5.862  (6) | 5.854  (7) | 5.795  (2) |
| *V* (Å^3^) | 309.681(1) | 302.946(1) | 299.233(1) | 296.378(3) | 293.044(3) | 290.395(2) | 288.058(3) | 285.478(2) | 283.084(4) | 280.347(4) | 277.054(4) | 276.158(4) | 268.944(3) |
| Z | 4 | | | | | | | | | | | | |
| Zn-S (Å) | 2.411 | 2.412 | 2.464 | 2.286 | 2.196 | 2.18 | 2.154 | 2.235 | 2.254 | 2.316 | 2.295 | 2.313 | 2.308 |
| Zn-O (Å) | 1.892 | 2.026 | 1.97 | 1.878 | 1.863 | 1.844 | 1.845 | 1.833 | 1.826 | 1.812 | 2.111 | 2.097 | 2.012 |
| S-Zn-S (deg) | 110.4 | 109.1 | 105.2 | 117.2 | 124.7 | 125.8 | 128.2 | 119.9 | 117.8 | 112.5 | 113.8 | 112.5 | 112.2 |
| O-Zn-O (deg) | 107.5 | 96.8 | 100 | 106.5 | 107.1 | 108.1 | 107.6 | 107.9 | 107.9 | 108.5 | 87.9 | 88.5 | 92.1 |
| *R*_wp_ (%) | 7.23 | 6.52 | 7.42 | 6.08 | 6.36 | 5.80 | 5.84 | 6.50 | 6.43 | 6.50 | 5.85 | 6.28 | 6.81 |
| *R*_p_ (%) | 4.40 | 4.49 | 4.65 | 4.01 | 4.43 | 4.07 | 4.01 | 4.43 | 4.36 | 4.41 | 3.99 | 4.19 | 4.48 |

**Table S2 Comparision of calculated and experimental Raman modes of BaZnOS**

| **Calculated modes (cm^-1^)** | **Experimental Raman peaks (cm^-1^)** | **Assignation** |
| --- | --- | --- |
| 64.7 | 71.2 | B_1_g |
| 85.5 | 90.2 | Ag |
| 114.5 | 124.0 | B_1_g |
| 180.2 | 191.2 | Ag |
| 272.3 | 284.2 | Ag |
| 284.6 | 300.0 | B_1_g |


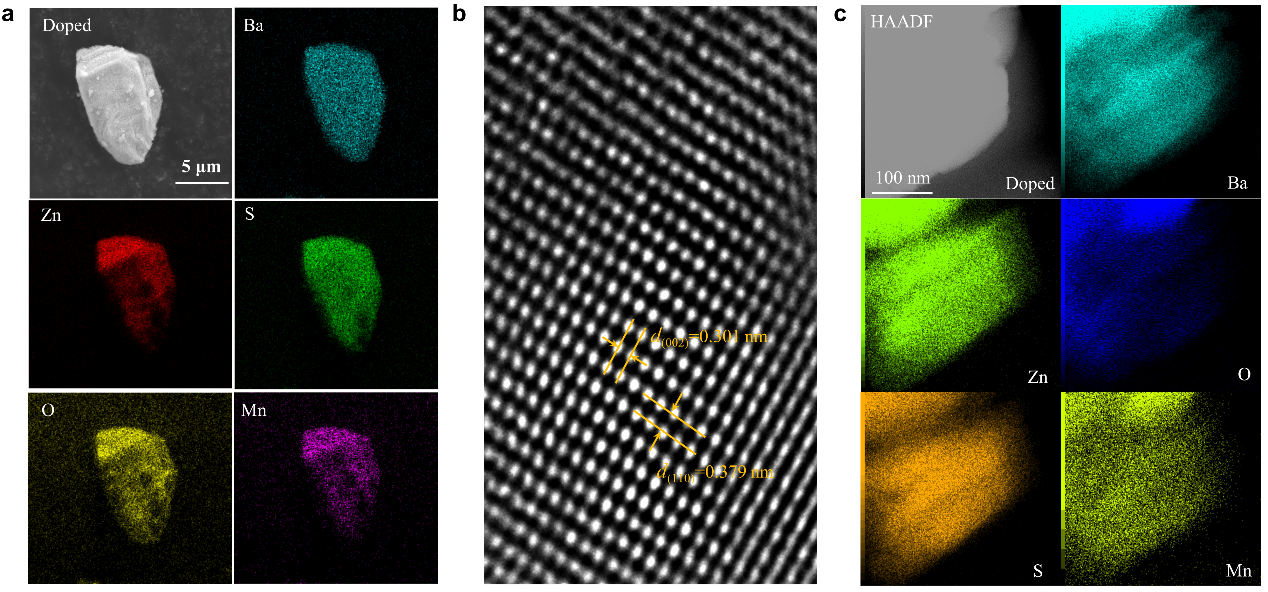


**Figure S1.** (a) Scanning electron microscope image and elemental mapping of a single BaZnOS: Mn^2+^ (1%) crystals. (b) High-resolution transmission electron (HRTEM) image displaying the (002) and (110) lattice fringes. (c) High-angle annular dark-field scanning transmission electron microscope (HAADF-STEM) image and corresponding compositional analysis of an individual particle. It shows that the Mn^2+^-doped BaZnOS have micron-sized grains with the homogeneous distribution of elemental Ba, Zn, O, S, and Mn in the BaZnOS matrix.


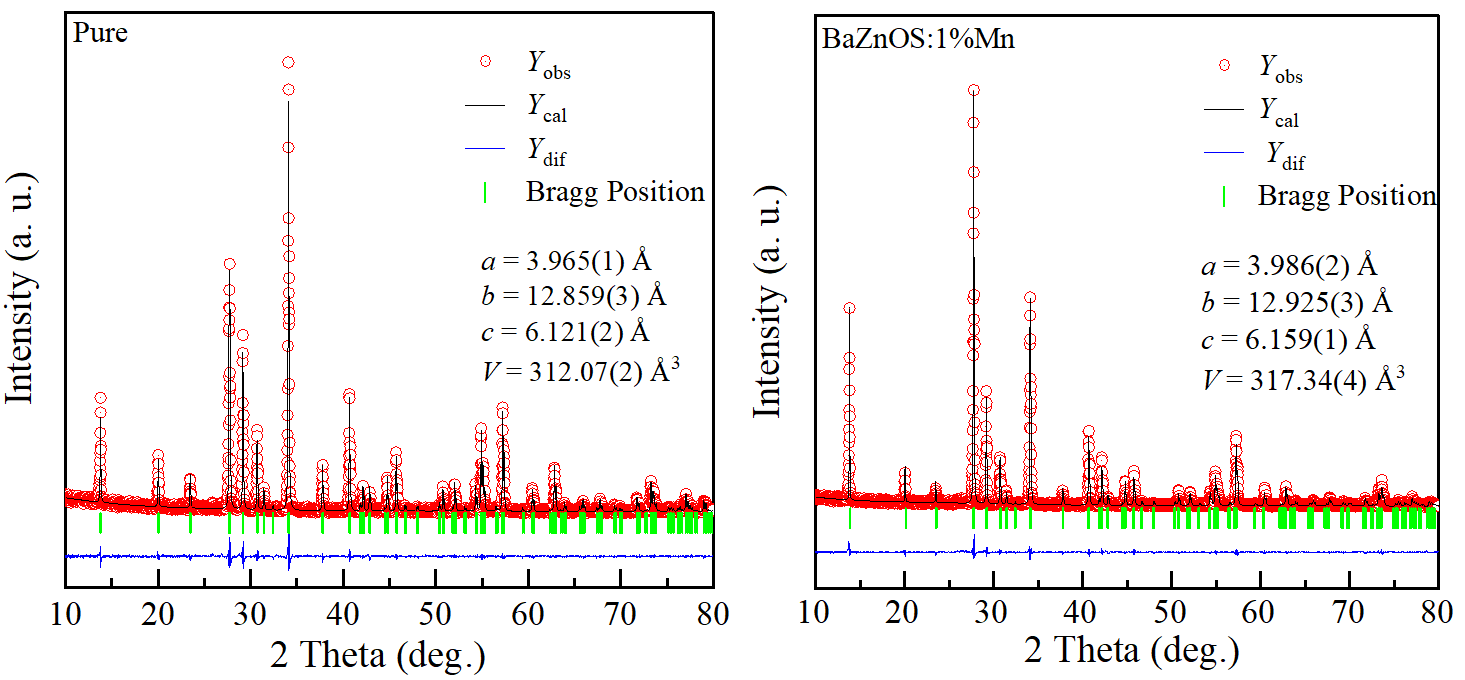


**Figure S2.** Refinement of the XRD pattern of undoped and 1%Mn-doped samples. BaZnOS crystallizes in the pure *Cmcm* orthorhombic structure with lattice parameters with *a* = 3.986(2), *b* = 12.925(3) and *c* = 6.159(1) for BaZnOS: 0.01Mn^2+^, and *a* = 3.965(1), *b* = 12.859(3) and *c* = 6.121(2) for pure BaZnOS. Mn^2+^ substitutes Zn^2+^ due to their similar ionic radii and chemical properties.


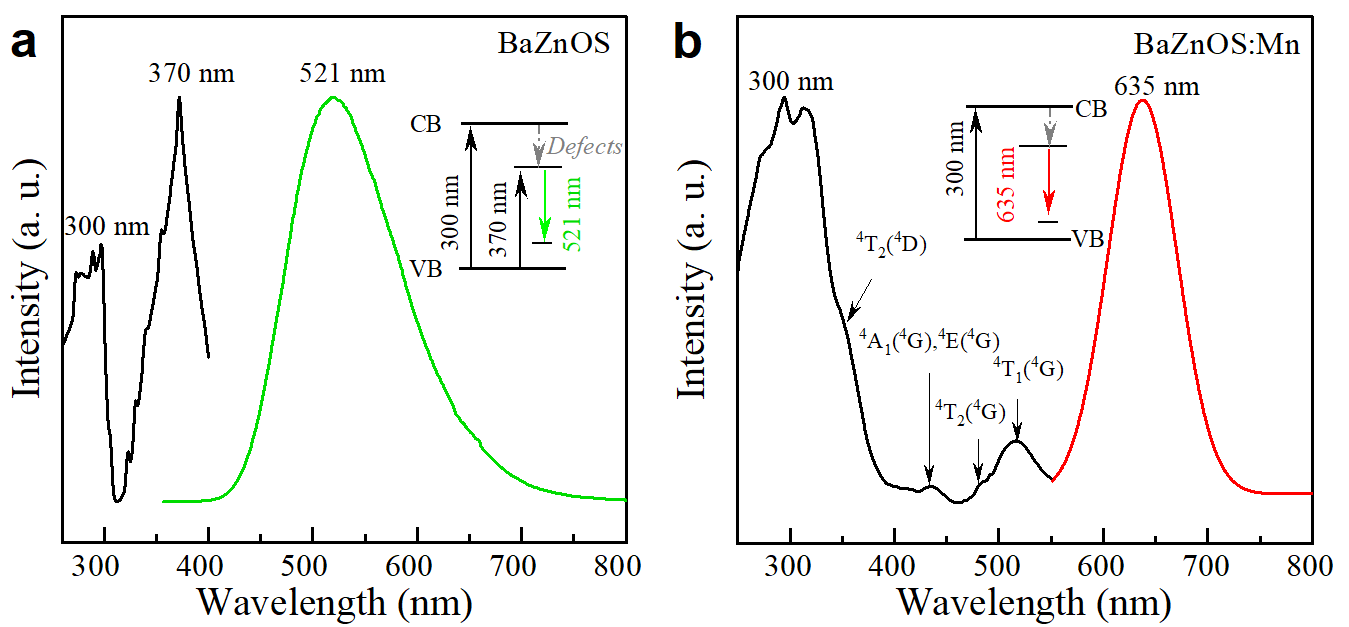


**Figure 3.** (a) Excitation and emission spectra of BaZnOS. (b) Excitation and emission spectra of Mn-doped BaZnOS. The weak bands of the excitation spectra in the range of 350-550 nm correspond to the d-d transitions of Mn^2+^.


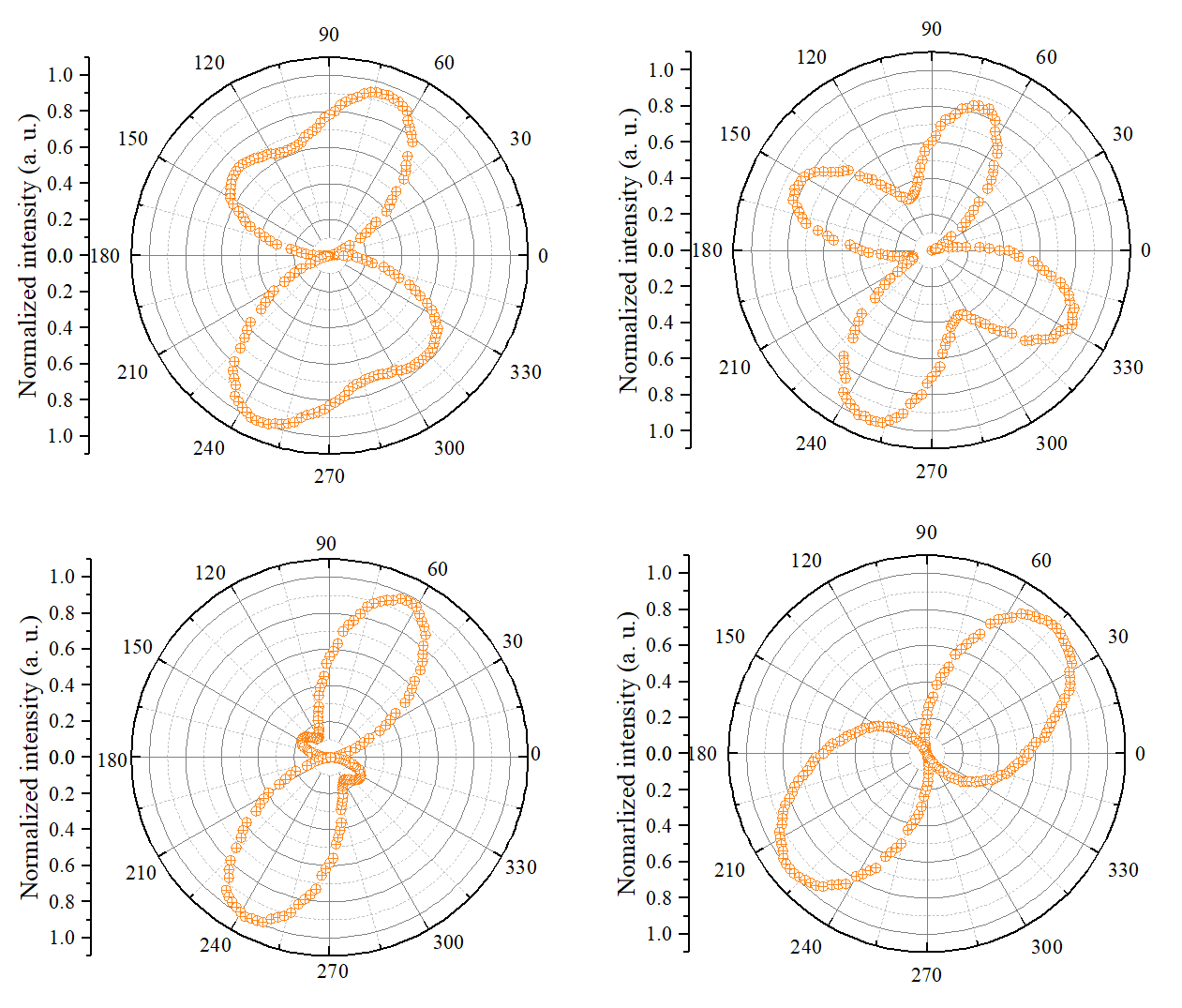


**Figure S4.** SHG signals of BaZnOS polycrystallines across multiple orientations at ambient conditions. It is noteworthy that the grain size of the samples is close to the micrometer laser spot scale, and thus, the polycrystalline samples exhibit polarization SHG properties similar to those of single crystals. Microscopic SHG tests show significant differences in the polarization-dependent response of different grains, and these anisotropic SHG responses originate from the orientation-dependent distribution of the microcrystals. This phenomenon provides important experimental evidence for the piezoelectricity in the ML generation.


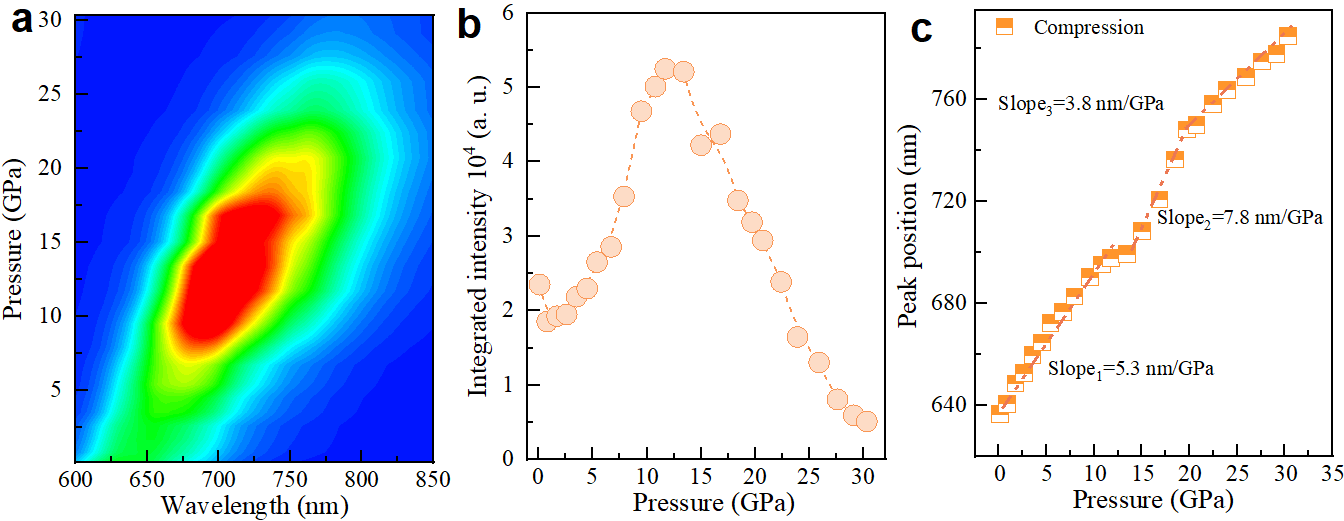


**Figure S5.** (a) 2D projection of PL spectra under high pressures. (b) PL intensity and (c) peak position as a function of pressure.


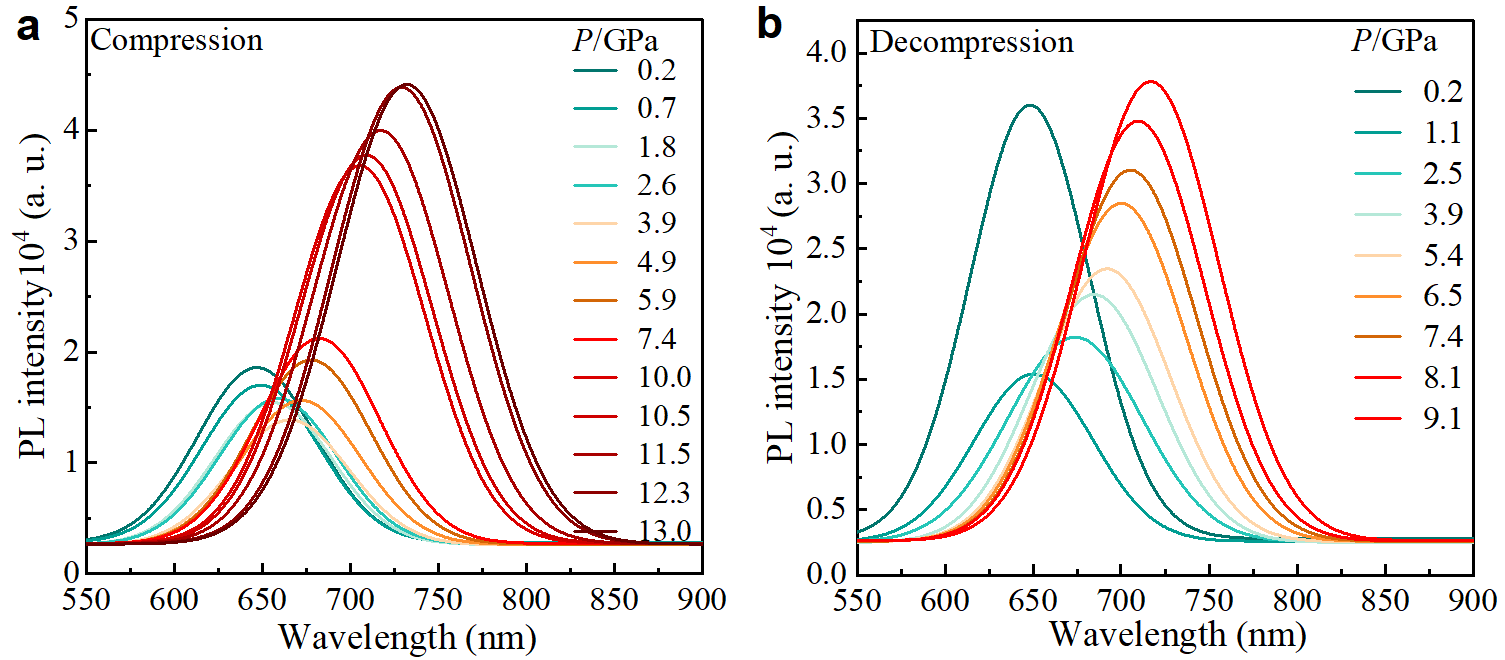


**Figure S6.** High-pressure PL spectra of BaZnOS: Mn^2+^ in the second run. (a) pressure-dependent PL spectra under compression and (b) decompression.


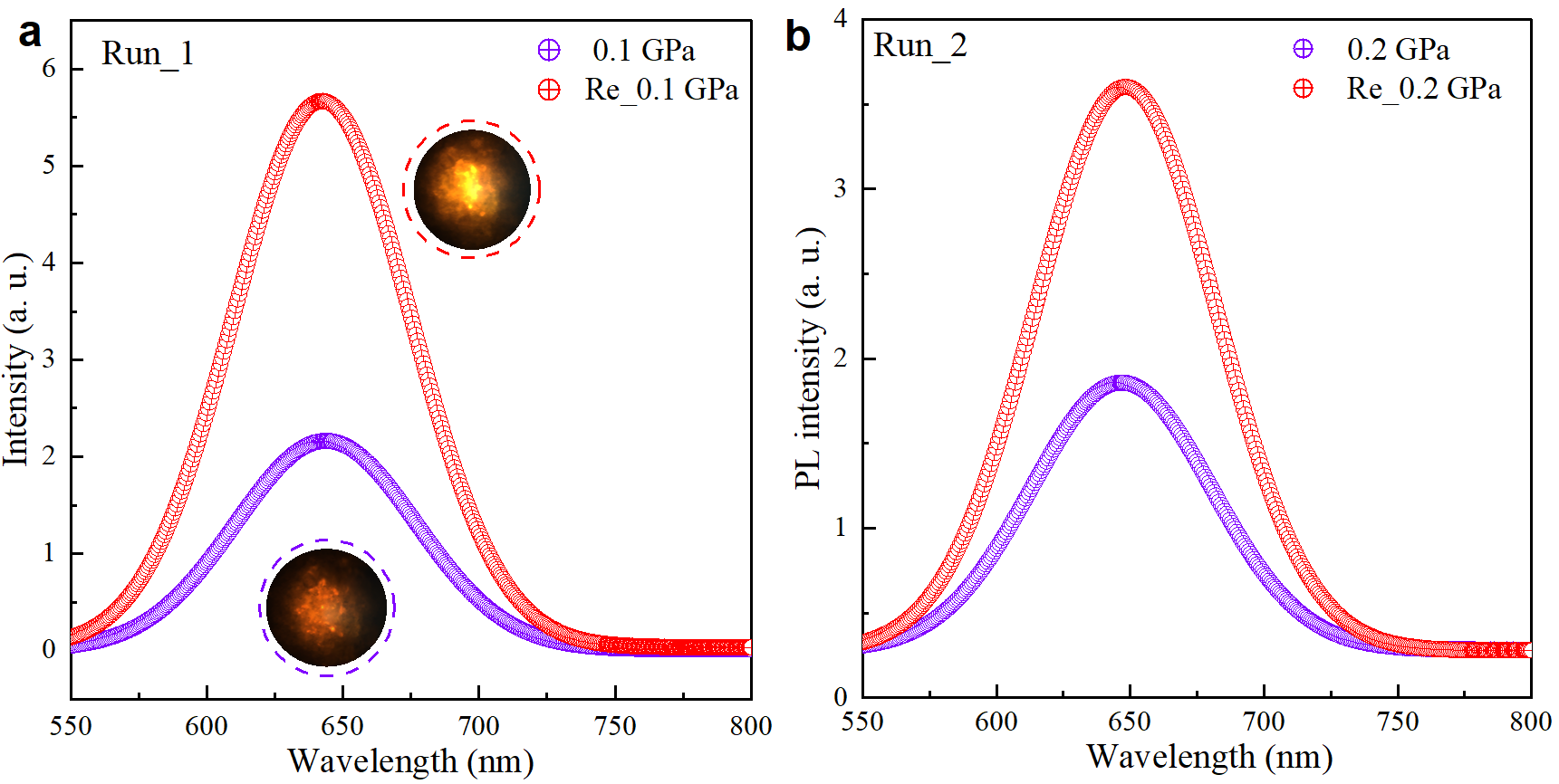


**Figure S7.** High-pressure treatment (a) in the first run and (b) in the second run. The enhancement of the PL intensity after pressure release may stem from irreversible local structural distortions.


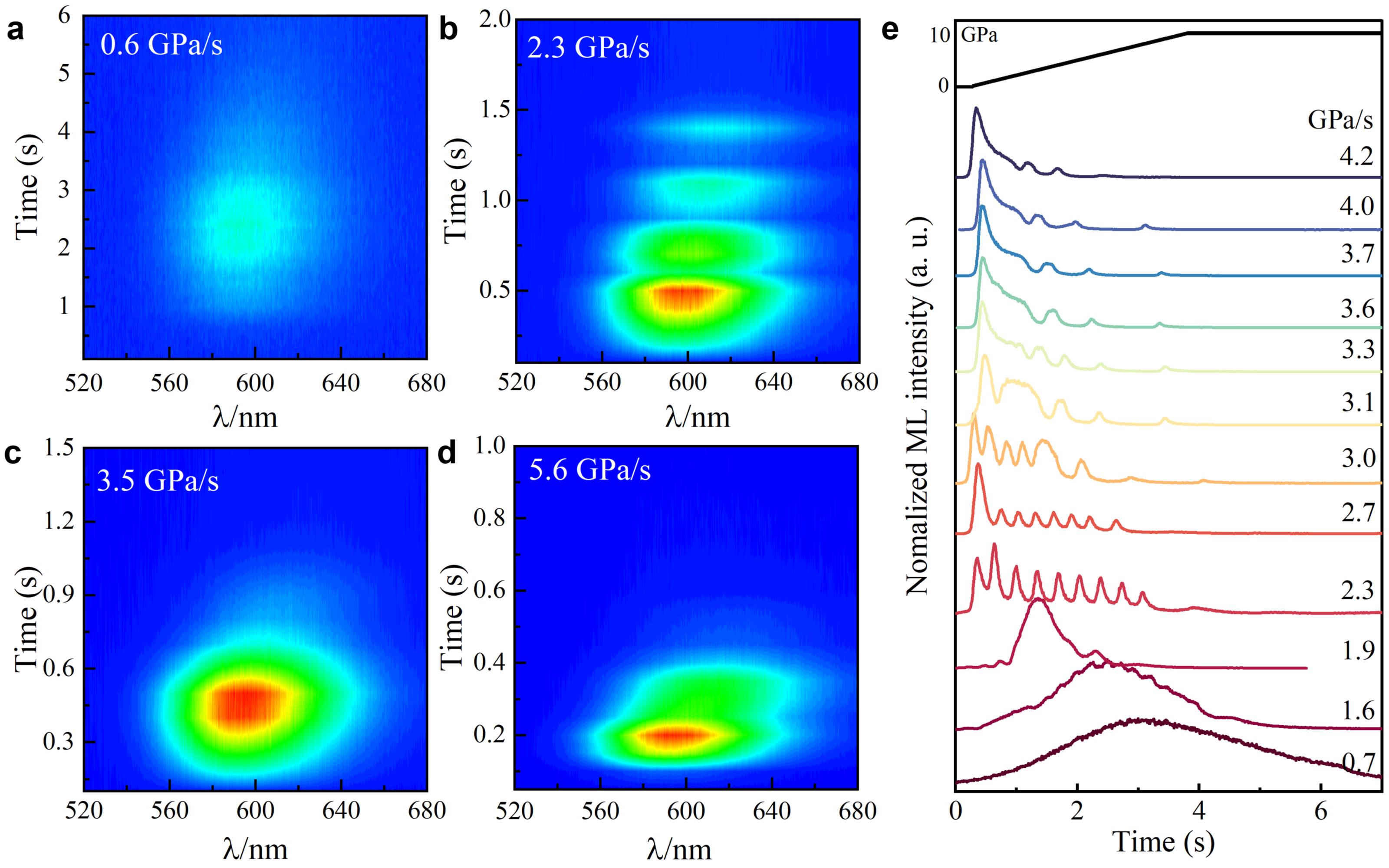


**Figure S8.** (a-d) Time-resolved ML spectra and (e) ML signal of BaZnOS: Mn^2+^ at different compression rates. It should be noted that the starting time for data collection is different at different rates in different runs. It means the ML emission may occur at different time at different rates.


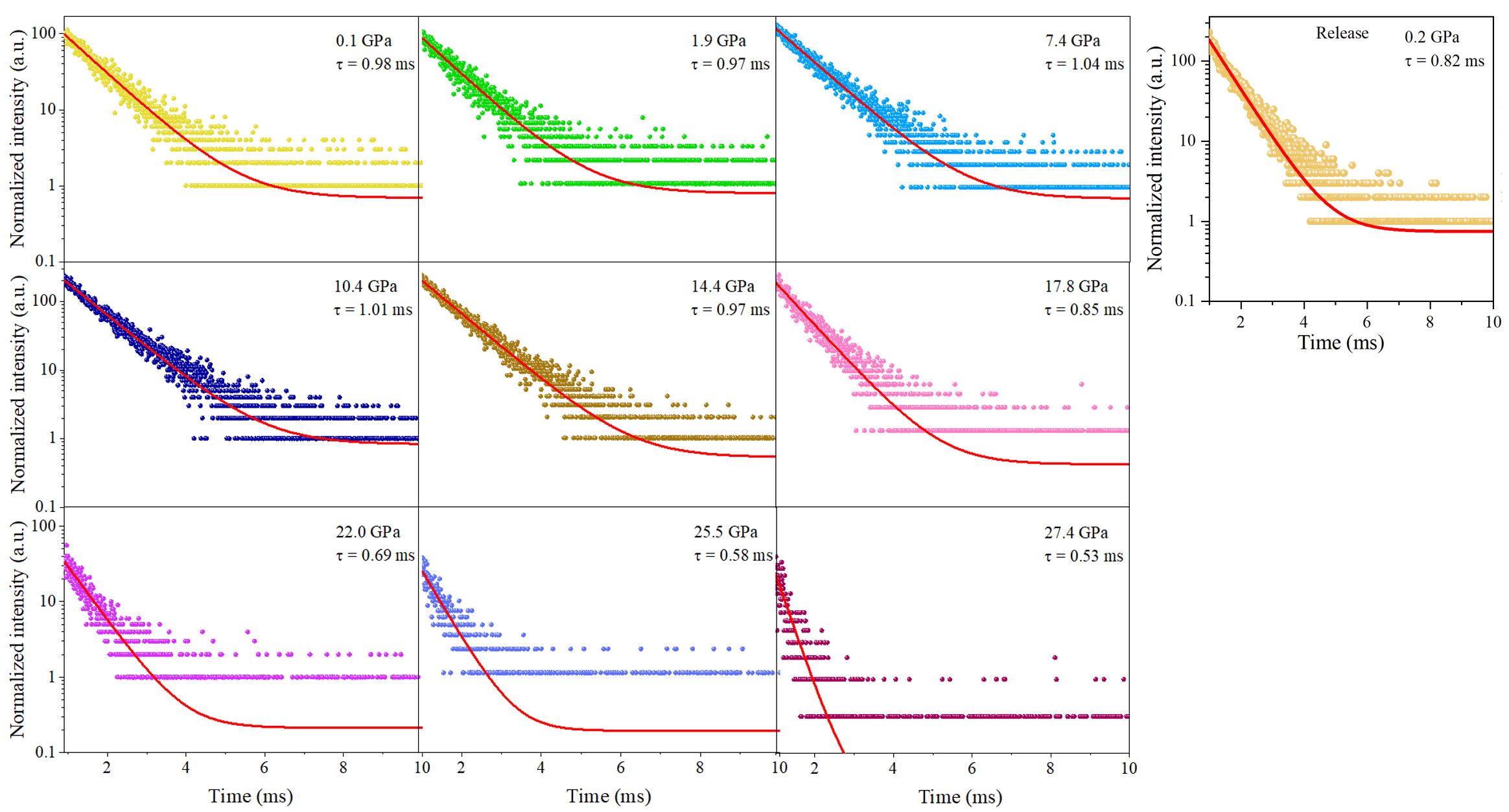


**Figure S9.** Pressure-induced decay curve and lifetime of PL for BaZnOS: Mn^2+^


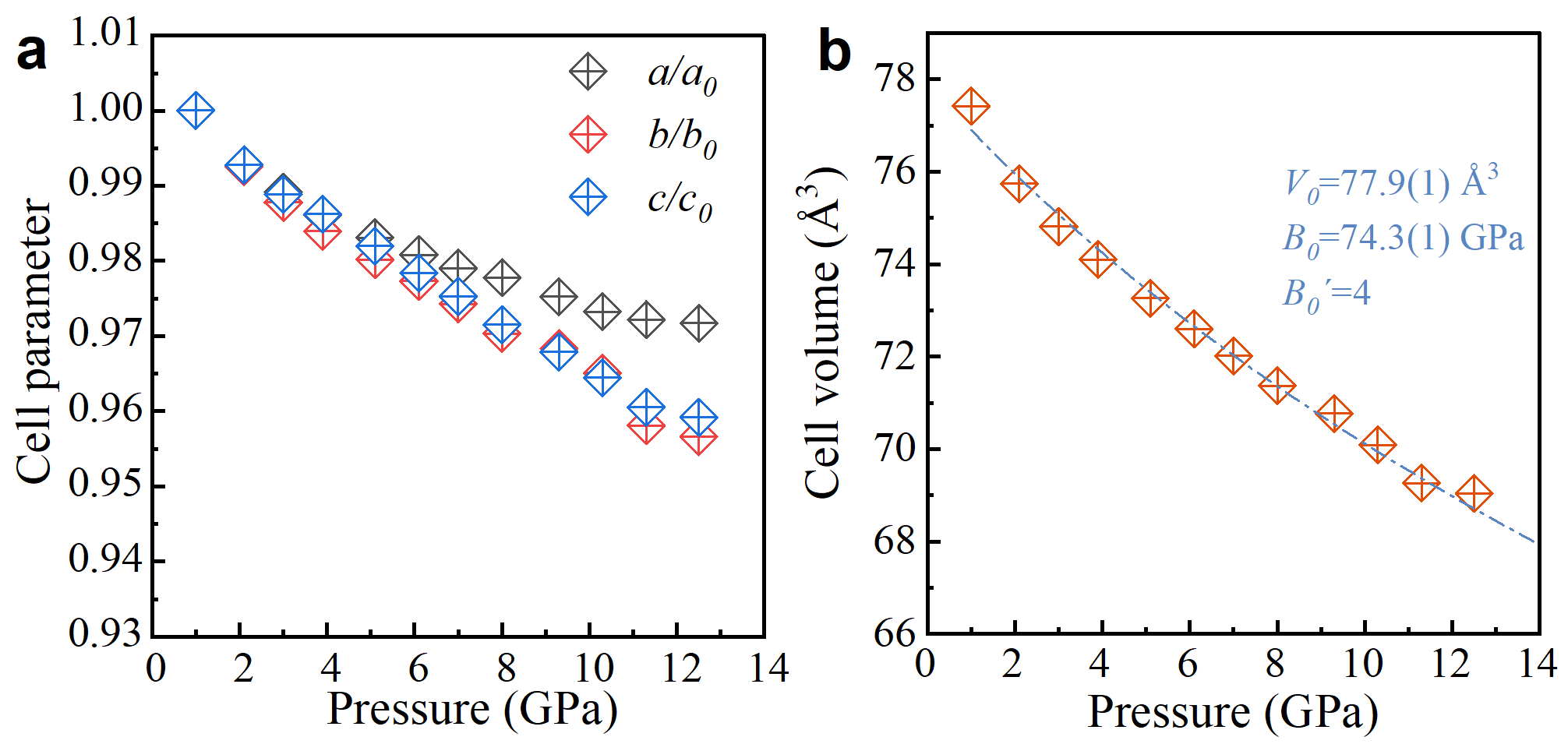


**Figure S10.** (a) Cell parameter and (b) cell volume as a function of pressure. The normalized lattice parameters *a*/*a_0_*, *b*/*b_0_*, *c*/*c_0_* and the cell volume of the BaZnOS decrease with increasing pressure and exhibit an anisotropic compression behavior. The experimental pressure−volume (*P-V*) data are fitted by the Birch−Murnaghan equation^[15]^

$$P\left( V \right)=\frac{3B_{0}}{2}\left[ \left( \frac{V_{0}}{V} \right)^{\frac{7}{3}}-\left( \frac{V_{0}}{V} \right)^{\frac{5}{3}} \right]\left\{ 1+\frac{3}{4}\left( B_{0}^{'}-4 \right)\left[ \left( \frac{V_{0}}{V} \right)^{\frac{2}{3}}-1 \right] \right\}$$

where *V_0_* is the unit cell volume at ambient pressure, *V* is the volume at pressure *P, B_0_* is the bulk modulus at ambient pressure, and *B_0_′* is a parameter for the pressure derivative. Fitting P-V data yields *V_0_* = 77.9(1) Å^3^, *B_0_* = 74.3(1) GPa, and *B_0_′* = 4 (fixed) for *Cmcm* structure. The observed results indicate the Mn^2+^ dopants have negligible influence on the structural stability and lattice compressibility of the host structure.


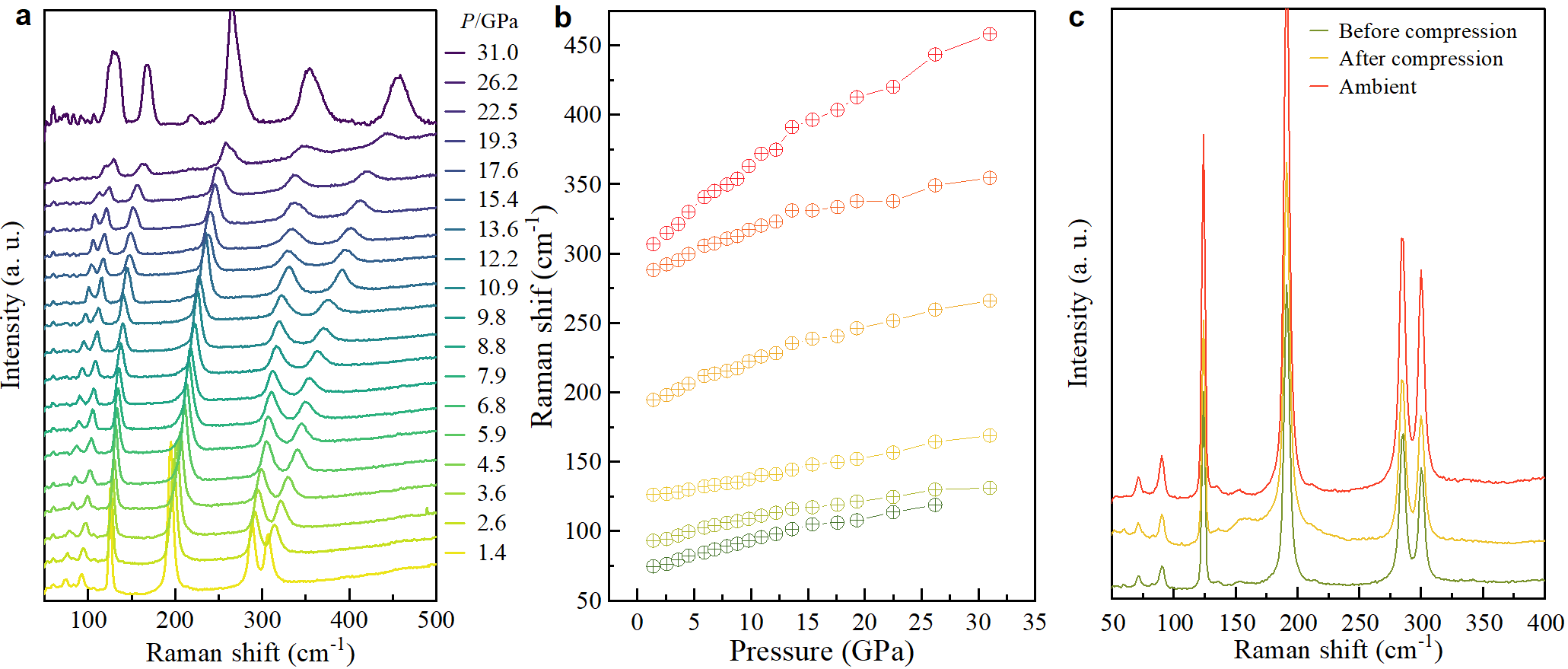


**Figure S11.** (a) High-pressure Raman spectra of BaZnOS excited by the 532 nm laser. (b) The Raman peaks as a function of pressure. (c) Comparison of Raman spectra before compression and after release of pressure.


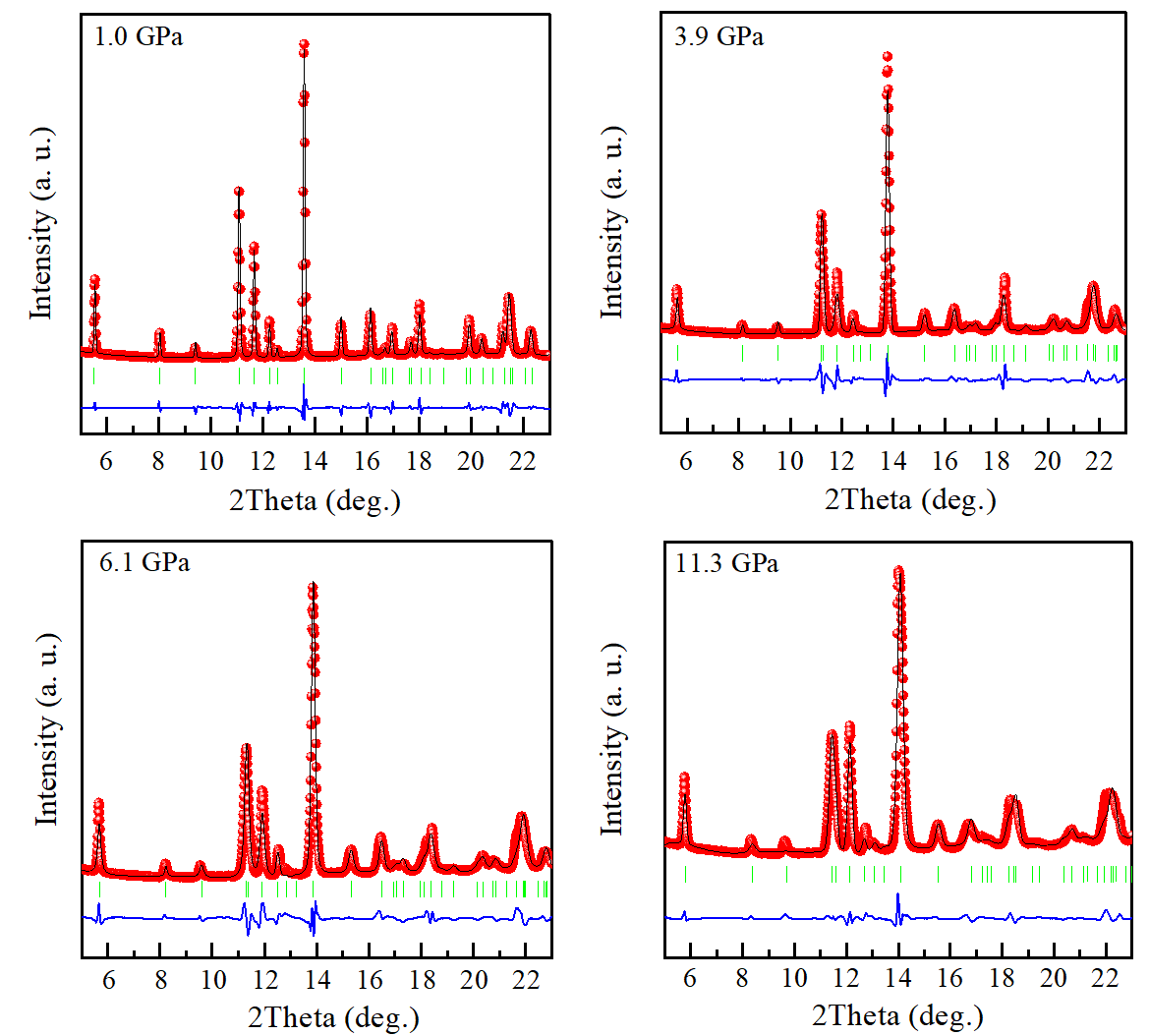


**Figure S12.** The refined XRD patterns of BaZnOS at selected pressures of 1.1 GPa, 3.9 GPa, 6.1 GPa, and 11.3 GPa, respectively.


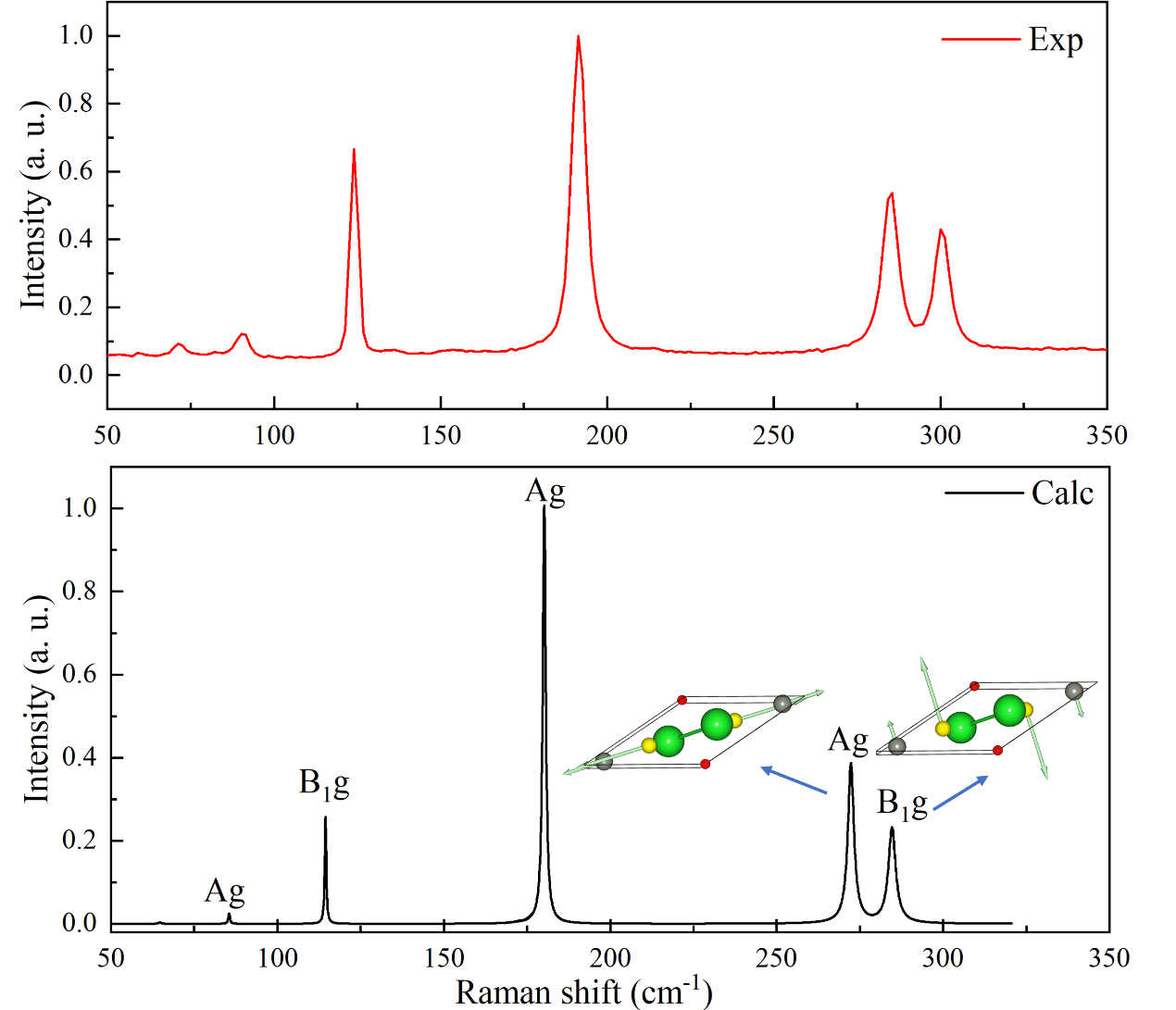


**Figure S13.** Comparison of calculated and experimental Raman spectra of BaZnOS.


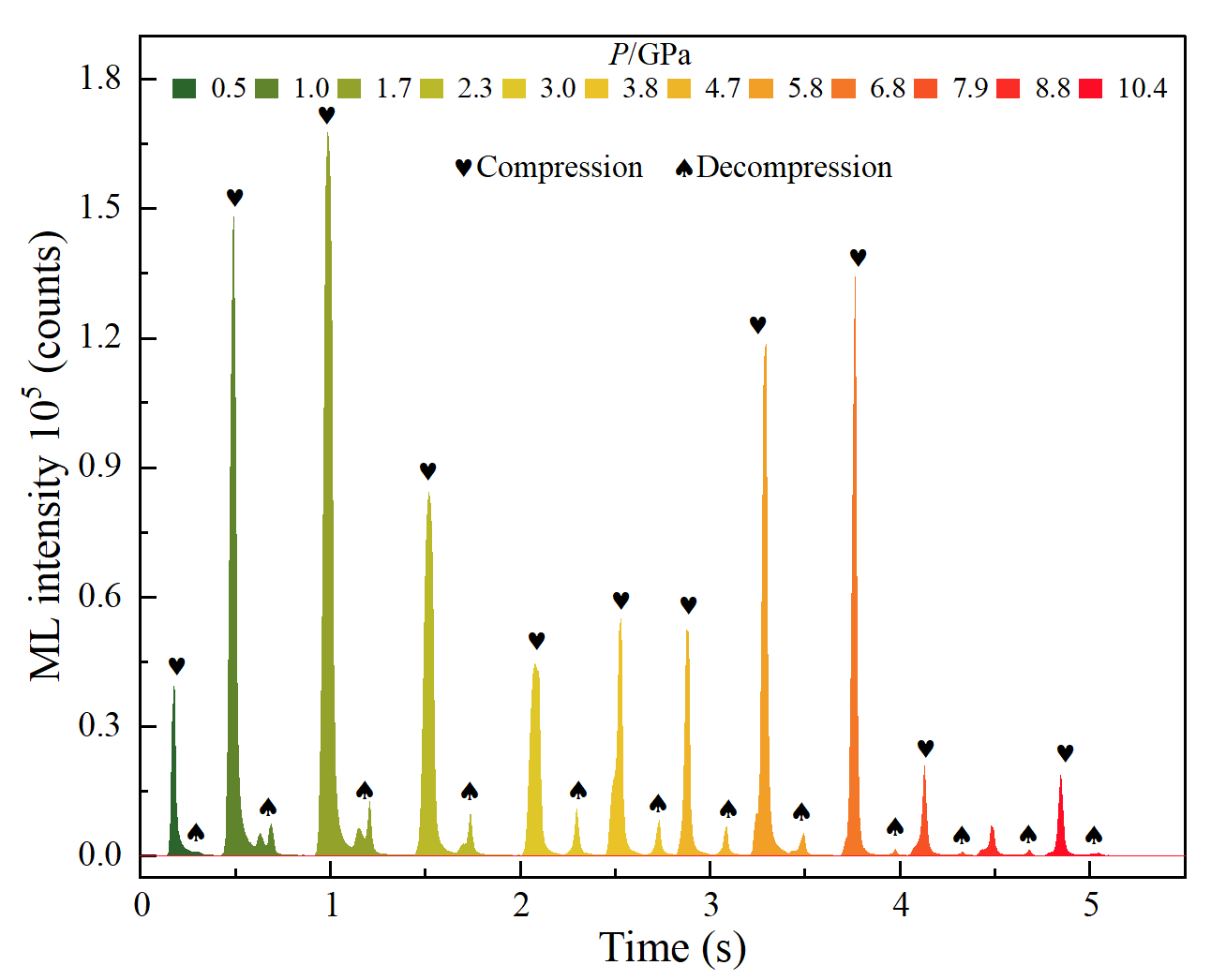


**Figure S14.** ML emission under rapid compression at different onset pressures.


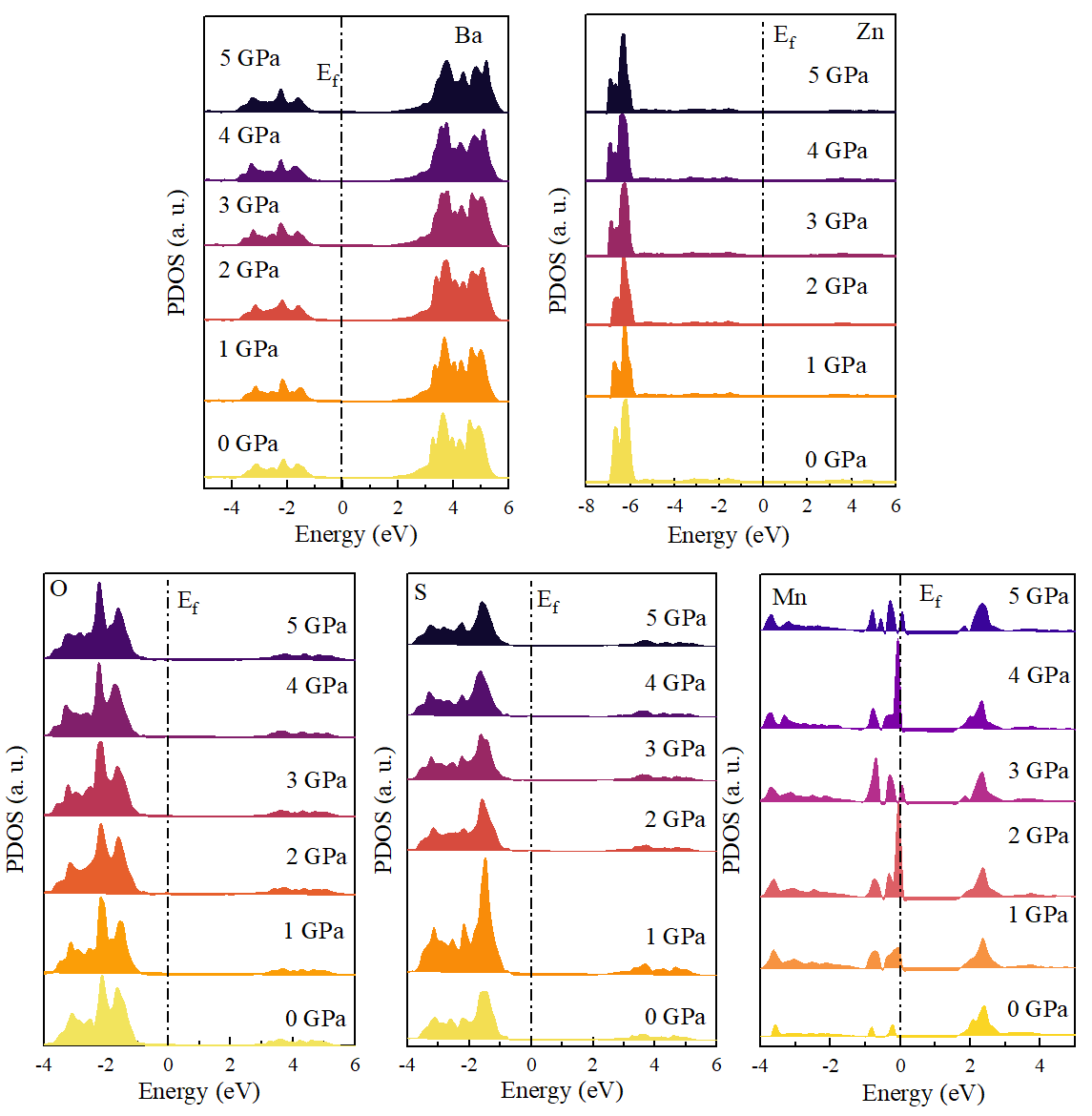


**Figure S15.** The PDOSs of Mn-doped, undoped BaZnOS, and elements (Ba, Zn, O, S, Mn) under different pressures.


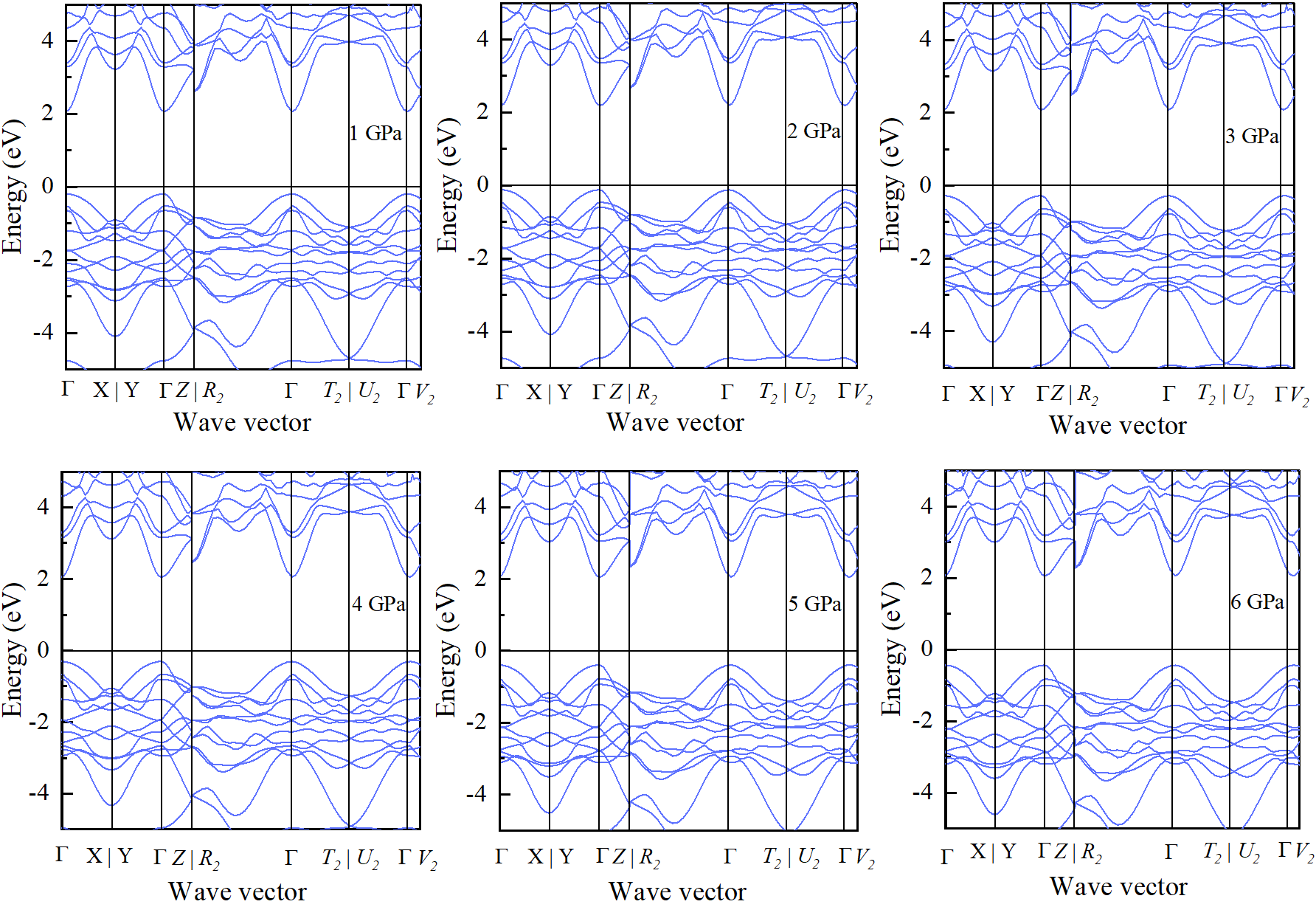


**Figure S16.** The band structure of BaZnOS under different pressures.

**Supplementary Movies**

**Supplementary references**

[1] S. Broadley, Z. A. Gál, F. Corà, C. F. Smura, S. J. Clarke. *Inorg. Chem.* **2005**, 44, 9092-9096.

[2] L. Guo, T. Wang, Q. Wang, W. Feng, Z. Li, S. Wang, P. Xia, F. Zhao, X. Yu. *Chem. Eng. J.* **2022**, 442, 136236.

[3] J.-A. Xu, H.-K. Mao, P.-M. Bell. *Science* **1986**, 232, 1404–1406.

[4] L. Dubrovinsky, N. Dubrovinskaia, V. B. Prakapenka, A. M. Abakumov. *Nat. Commun.* **2012**, 3, 1163.

[5] H.-K. Mao, J.-A. Xu, P.-M. Bell. *J. Geophys. Res.* **1986**, 91, 4673–4676.

[6] C. Prescher, V. B. Prakapenka. *High Press. Res.***2015**, 35, 223–230.

[7] B. H. Toby. *J. Appl. Crystallogr.* **2001**, 34, 210–213.

[8] S. K. Kurtz, T. T. Perry. *J. Appl. Phys.* **1968**, 39, 3798–3813.

[9] G. Kresse. J. Hafner. *Phys. Rev. B* **1993**, 47(1), 558-561.

[10] G. Kresse, J. Furthmüller. *Phys. Rev. B* **1996**, 54, 11169-11186.

[11] G. Kresse, D. Joubert. *Phys. Rev. B* **1999**, 59, 1758-1775.

[12] J. P. Perdew, K. Burke, M. Ernzerhof. *Phys. Rev. Lett.* **1996**, 77, 3865.

[13] G. Kresse, J. Furthmüller. *Comput. Mater. Sci.* **1996**, 6, 15-50.

[14] A. Togo. *J. Phys. Soc. Jpn.* **2022**, 92, 012001.

[15] F. Birch. *Phys. Rev.* **1947**, 71, 809–824.
